# Molecular Diversity via Tetrasubstituted Alkenes Containing a Barbiturate Motif: Synthesis and Biological Activity

**DOI:** 10.3390/molecules25245868

**Published:** 2020-12-11

**Authors:** Ahmed Al-Sheikh, Masuma Begum, Bian Zhang, Richard A. Lewis, Nicholas E. E. Allenby, Paul G. Waddell, Bernard T. Golding

**Affiliations:** 1 Faculty of Pharmacy, University of Petra, P.O. Box 961343, Amman 11196, Jordan; 2 BiBerChem Research Ltd., The Biosphere, Draymans Way, Newcastle Helix, Newcastle upon Tyne NE4 5BX, UK; bmasuma77@googlemail.com (M.B.); b.zhang@biberchem.com (B.Z.); 3 Demuris Ltd., The Biosphere, Draymans Way, Newcastle Helix, Newcastle upon Tyne NE4 5BX, UK; richard.lewis@demuris.com (R.A.L.); nick.allenby@demuris.co.uk (N.E.E.A.); 4 Chemical Crystallography Unit, School of Natural & Environmental Sciences, Bedson Building, Newcastle University, Newcastle upon Tyne NE1 7RU, UK; paul.waddell@ncl.ac.uk; 5 School of Natural & Environmental Sciences, Bedson Building, Newcastle University, Newcastle upon Tyne NE1 7RU, UK

**Keywords:** molecular diversity, barbiturate template, antifungal, antibacterial, trifluoroethanol

## Abstract

The synthesis of a molecularly diverse library of tetrasubstituted alkenes containing a barbiturate motif is described. Base-induced condensation of *N*^1^-substituted pyrimidine-2,4,6(1*H*,3*H*,5*H*)-triones with 5-(bis(methylthio)methylene)-2,2-dimethyl-1,3-dioxane-4,6-dione gave 3-substituted 5-(methylthio)-2*H*-pyrano[2,3-*d*]pyrimidine-2,4,7(1*H*,3*H*)-triones (‘pyranopyrimidinones’), regioselectively. A sequence of reactions involving ring-opening of the pyran moiety, displacement of the methylthio group with an amine, re-formation of the pyran ring, and after its final cleavage with an amine, gave tetrasubstituted alkenes (3-amino-3-(2,4,6-trioxotetrahydropyrimidin-5(2*H*)-ylidene)propanamides) with a diversity of substituents. Cleavage of the pyranopyrimidinones with an aniline was facilitated in 2,2,2-trifluoroethanol under microwave irradiation. Compounds were tested against *Escherichia coli*, *Staphylococcus aureus*, the yeast *Schizosaccharomyces pombe,* and the pathogenic fungus *Candida albicans*. No compounds exhibited activity against *E. coli*, whilst one compound was weakly active against *S. aureus*. Three compounds were strongly active against *S. pombe*, but none was active against *C. albicans*.

## 1. Introduction

Invasive fungal infections (IFIs) cause the deaths of about 1.5 million people every year [1] and are particularly prevalent in intensive care units, affecting elderly and/or immunocompromised patients. As with antibacterial agents [2], resistance to current antifungal drugs is a significant clinical problem [3]. The relatively few clinically useful antifungal drugs include inhibitors of ergosterol biosynthesis (e.g., azoles), the polyene macrolide antibiotics compromising ergosterol function (e.g., natamycin), and echinocandins (e.g., caspofungin) affecting cellular β-glucan synthesis. These compounds suffer from several liabilities, including fungal resistance (azoles, polyenes), lack of broad-spectrum activity (echinocandins), and toxicity (polyenes). There is a clinical need for improved antifungals, which either represent new structural classes or are novel members of existing classes [4].

**Figure 1:**
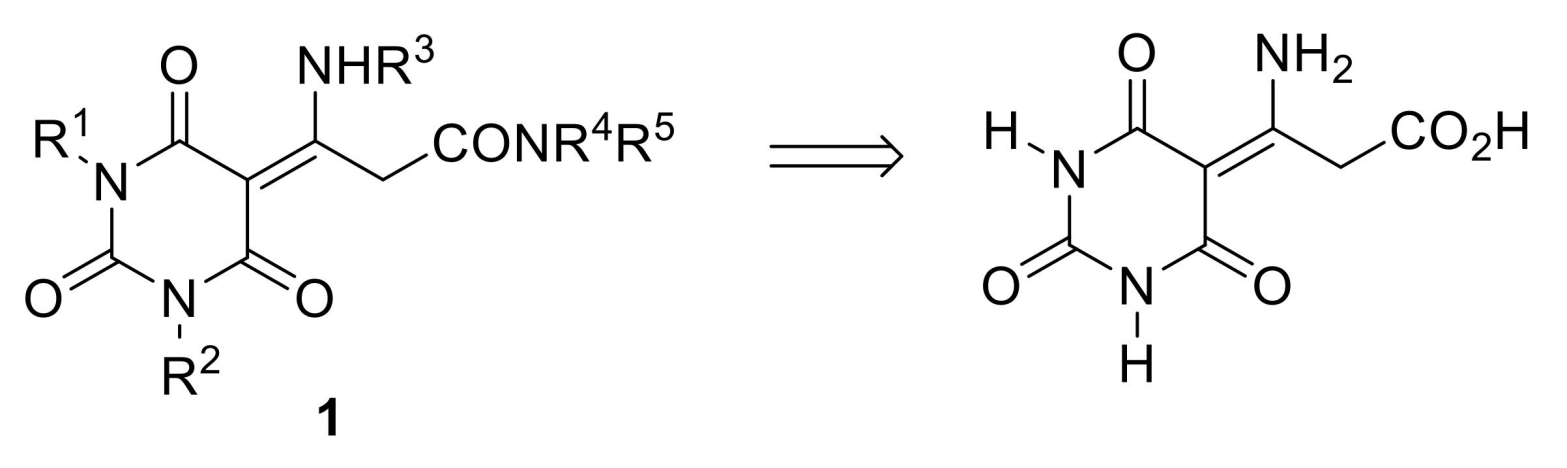
Derivatives **1** of 3-amino-3-(2,4,6-trioxotetrahydropyrimidin-5(2*H*)-ylidene)propanoic acid.

The creation of molecular diversity [5,6] and privileged scaffolds [7] leading to new ‘drug-like molecules’ is an important direction of research with the promise that entirely new agents will arise. We have found a strategy for the construction of tetrasubstituted alkenes (derivatives **1** of 3-amino-3-(2,4,6-trioxotetrahydropyrimidin-5(2*H*)-ylidene)propanoic acid, Figure 1) containing a barbiturate motif. These compounds were obtained via intermediate 2*H*-pyrano[2,3-*d*]pyrimidine-2,4,7(1*H*,3*H*)-triones. The compounds described have been tested against selected organisms, revealing promising antifungal activity.

Our strategy for the construction of tetra-substituted alkene **1** permits the systematic incorporation of structural units from amine nucleophiles, giving a variety of products. This approach enables molecular diversity to be explored by judicious selection of starting materials leading to structural variations at up to five points of appendage diversity within two distinct scaffolds. A focussed library of compounds has been generated based on the structure of derivative **1** that varies the group at each of R^1^, R^2^, R^3^, R^4,^ and R^5^ with respect to the parameters of group size and polarity. Jeong and Moloney [8] and others [9,10] have recently described analogous compounds with an antibacterial activity that are dissimilar in structure from those described here and accessed synthetically in a different manner. Notably, novel barbituric acids exhibited antibacterial activity, especially against resistant Gram-positive strains, such as *Staphylococcus aureus* (methicillin-resistant, MRSA), *Enterococcus faecalis* (vancomycin-susceptible, VSE), *E. faecium* (vancomycin-resistant, VRE), and *Streptococcus pneumonia* (multidrug-resistant, MDRSP) [8]. A major difference from the previous studies is that our synthetic approach directly produces esters and amides corresponding to structure **1**, avoiding the parent carboxylic acid, which is expected to be prone to decarboxylation.

## 2. Results

### 2.1. Synthesis of Pyranopyrimidinones **2a**–**e**

**Scheme 1:**
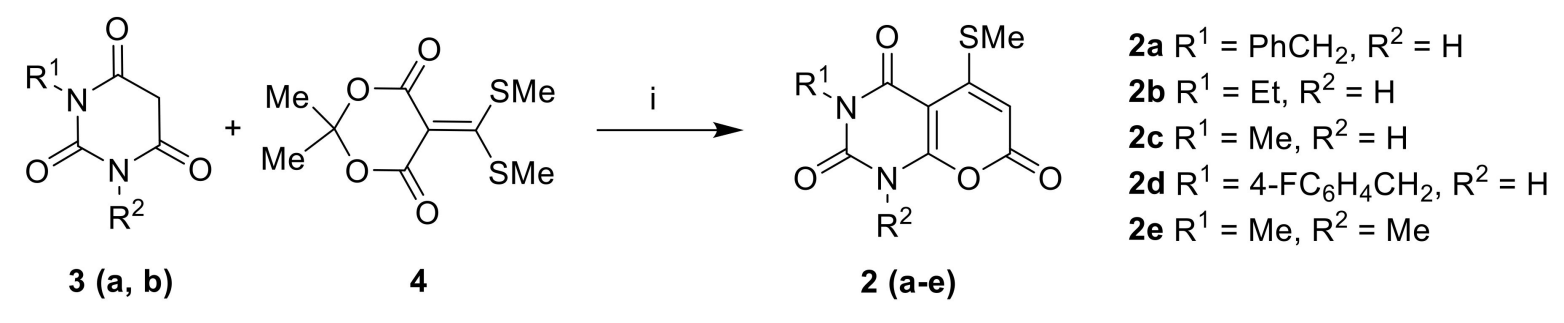
Synthesis of pyranopyrimidinones **2a**–**e**. *Reagents and conditions*: (i) heat at reflux in pyridine (py) for 1 h.

**Figure A1:**
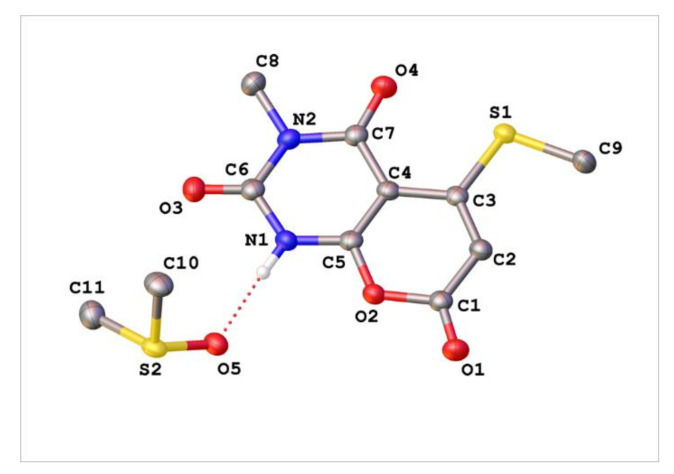
Crystal structure of compound **2c** (DMSO solvate). In this figure and Figure A2, Figure A3, Figure A4 and Figure A5, ellipsoids were rendered at the 50% probability level. Hydrogen atoms have been omitted for clarity except for those bound to heteroatoms.

Regioselective condensation between readily available barbiturate derivatives (substituted pyrimidine-triones, **3**) and compound **4** [11,12] gave pyranopyrimidinones **2** (Scheme 1). When R^1^ = alkyl (Me **2c**, Et **2b**) or substituted alkyl (benzyl **2a**, 4-fluorobenzyl **2d**) and R^2^ = H, one regioisomer was formed almost exclusively, as validated by crystal structure analysis for compound **2c** (Appendix A, Figure A1). Reactions of compound **4** with amines displacing one or both methylthio groups proceed by Michael addition, followed by the elimination of methanethiol [11,13,14].

### 2.2. Synthesis of Alkenes **5a**–**5l**

**Scheme 2:**
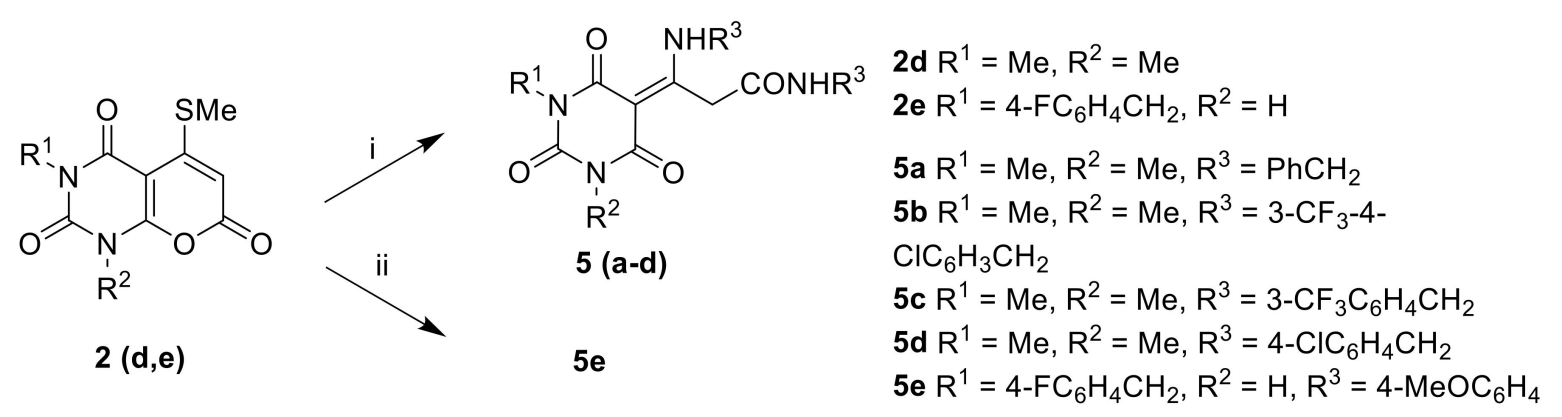
Synthesis of alkenes **5a**–**e**. *Reagents and conditions:* (i) benzylamine or substituted benzylamine, heat at reflux in EtOH for 1 h, (ii) 4-methoxyaniline, heat at 150 °C in dimethyl sulfoxide (DMSO) for 2 h.

**Figure A2:**
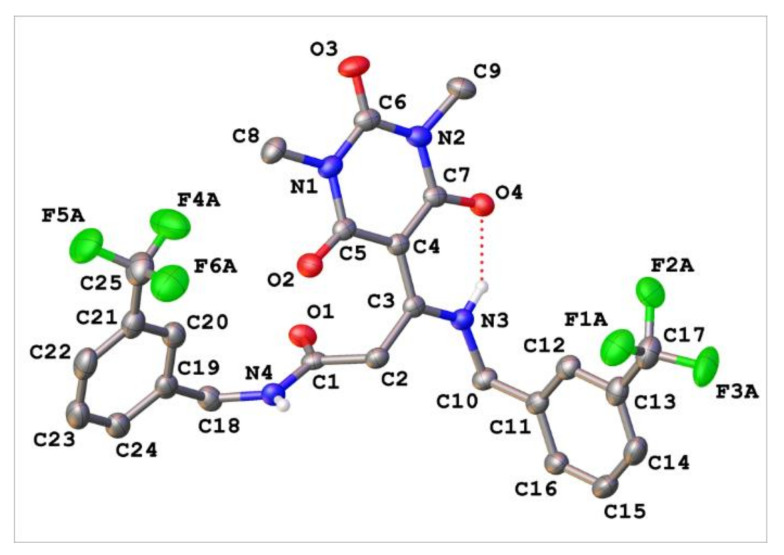
Crystal structure of compound **5c**.

Heating **2e** with benzylamines in ethanol gave alkenes **5a**–**d** (Scheme 2). Crystal structure analysis of **5c** confirmed its structure (Appendix A, Figure A2), which shows there is an intramolecular hydrogen bond between the amino NH and neighboring pyrimidine trione carbonyl group. The amide proton of **5c** forms an intermolecular hydrogen bond to the amide carbonyl of an adjacent molecule (See Appendix A for a fuller description of this structure).

**Scheme 3:**
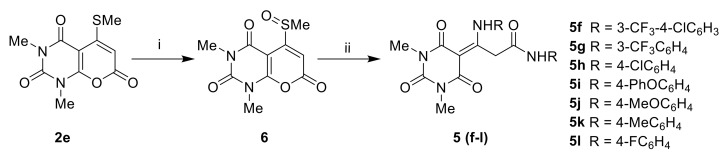
Synthesis of alkenes **5f**–**l.** *Reagents and conditions:* (i) *m*-chloroperbenzoic acid, dichloromethane (DCM) stirring at room temperature (r.t.) for 2 h, (ii) heat at reflux in EtOH for 1 h.

With the less nucleophilic anilines, it was preferable to proceed via oxidation of **2e** by *m*-chloroperbenzoic acid to **6**, which on heating with an aniline derivative in ethanol gave alkenes **5f**–**l** (Scheme 3).

### 2.3. Synthesis of Compounds **8a**–**f**, **9a**–**f** and **10a**–**q**

**Scheme 4:**
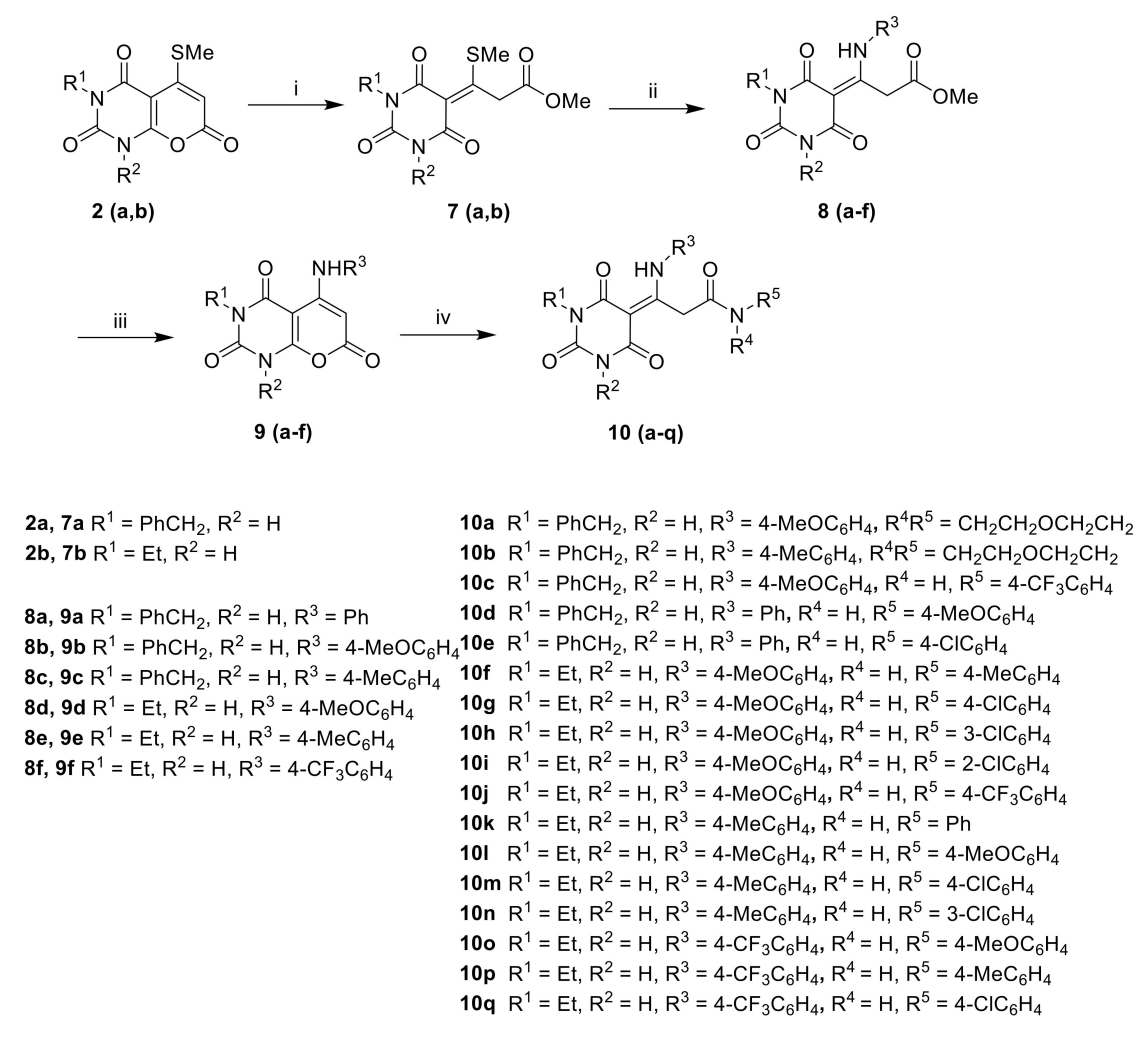
Synthesis of alkenes **10a**–**q**. *Reagents and conditions*; (i) NaOMe, heat at reflux in MeOH overnight, (ii) R^3^NH_2_, heat at reflux in EtOH overnight, (iii) aq. NaOH, heat at 70 °C in MeOH for 1 h, (iv) HNR^4^R^5^, μW at 130 °C in TFE for 1 h.

**Figure A3:**
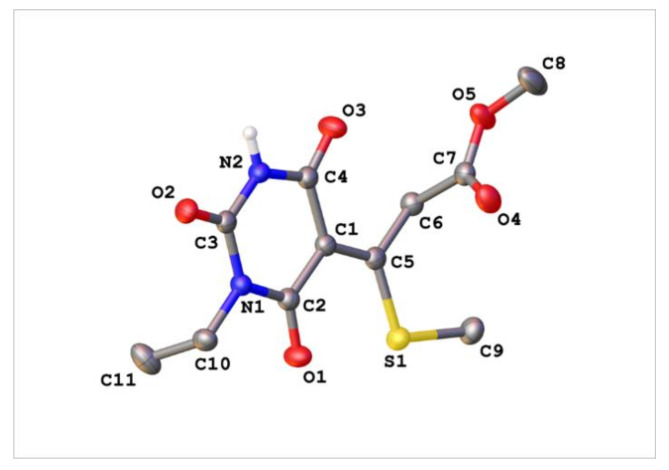
Crystal structure of compound **7b**.

**Figure A4:**
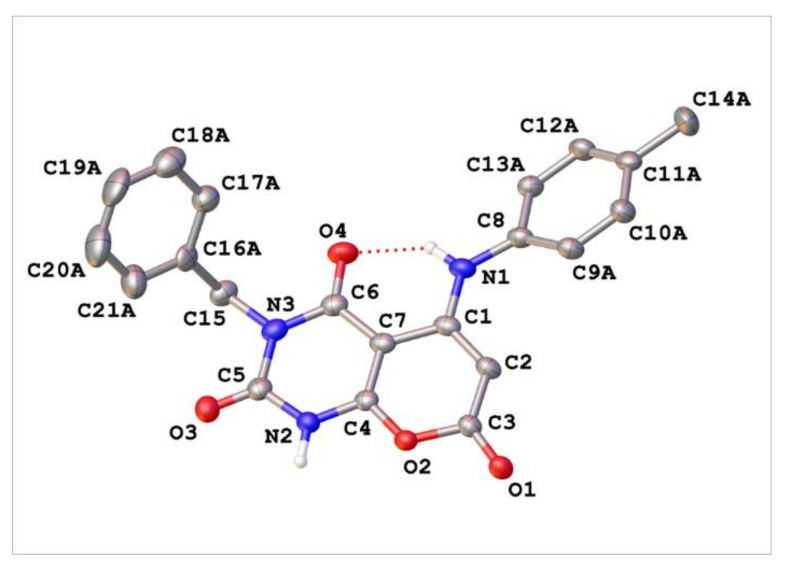
Crystal structure of compound **9c**.

**Figure A5:**
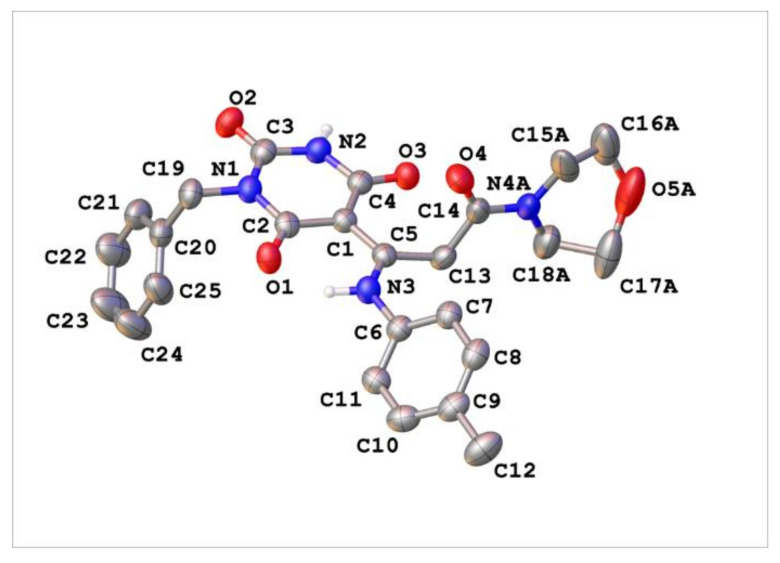
Crystal structure of compound **10b**.

Given the failure to effect stepwise substitution of the methylthio group and cleavage of the pyrone moiety when **2d** or **2e** were treated with amines (cf. Scheme 2), a different approach was adopted. Thus, heating compound **2a** or **2b** with sodium methoxide in methanol afforded methyl esters (**7a,b**). Compound **7** was reacted with an amine in ethanol to give alkenes **8a**–**f**. Heating **8** with NaOH in methanol gave pyranopyrimidinones **9a**–**f**. A solution of **9** and the corresponding amine in 2,2,2-trifluoroethanol (TFE) was subjected to microwave irradiation to give the target alkenes **10a**–**q** (Scheme 4). The structures and purities of the newly synthesized compounds were confirmed by ^1^H/^13^C-NMR spectroscopy and liquid chromatography-mass spectrometry, with further validation by crystal structure analysis in some cases (Appendix A, Figure A3, Figure A4 and Figure A5).

The structures of **7b**, **9c,** and **10b** were determined by single-crystal X-ray crystallography (Figure A3, Figure A4 and Figure A5; see Appendix A for a fuller description of the structures) and were representative of the compound groups **7**, **9,** and **10,** respectively. As observed with **5c**, an amino (NHR^3^) to carbonyl intramolecular hydrogen bond was apparent in **9c** and **10b**, forming a 6-membered ring. The configuration of the C=C bond in crystalline **7b** was the *Z* isomer, arising from nucleophilic attack by methoxide on the pyrone ring carbonyl of **2b**. Although compound **10b** exhibited *Z* configuration in its crystal structure, the ^1^H-NMR in *d*_6_-dimethylsulfoxide at ambient temperature showed that this compound and its congeners are mixtures of *E* and *Z* isomers in solution, the resonances from which coalesced at 90 °C.

### 2.4. Biological Assays

**Figure A6:**
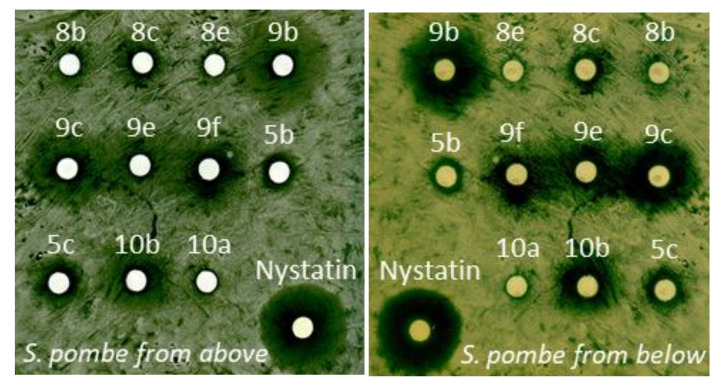
Zones of inhibition for *S. pombe* spread on agar plates and inoculated at specific points with test compounds; the plate was viewed from above (left) and below (right).

Disc bioassays were performed on compounds **5a**–**l**, **8a**–**f**, **9a**–**f,** and **10a**–**q** against the Gram-negative bacterium *Escherichia coli* (DH5α), the Gram-positive bacterium *Staphylococcus aureus* (RN4220), the unicellular eukaryotic yeast *Schizosaccharomyces pombe* (SAK950) and the human pathogenic fungus *Candida albicans* ATCC 90028. The most interesting results against *S pombe* are summarised in Table 1. Scanned images of bioassay plates are shown in Appendix B (Figure A6).

As shown in Table 1 and Figure A6, compounds **9b**, **9c,** and **10b** were strongly active against *S pombe*, with **9b** being comparable to nystatin. Significant inhibition was also observed for compounds **5b**, **5c**, **8b**, **8c**, **8e**, **9e**, **9f,** and **10a**. However, compounds **8a**, **8d**, **8f**, **9a**, **9d**, **5a**, **5d**–**l**, and **10c**–**q** were all inactive. None of the compounds was active against *C. albicans.* No compounds exhibited activity against *E. coli,* and only one compound (**10a**) was active, albeit weakly, against *S. aureus* (data not shown).

## 3. Discussion

An efficient route (Scheme 4) is described to afford a diverse library of alkenes **10** via esters **8** and pyranopyrimidinones **9**. Compounds derived from **8** were readily obtained from **4**. The route enables systematic variation of up to five substituents (R^1^, R^2^, R^3^, R^4^, and R^5^, compared to structures in **10**). Experimental details for the synthesis of all compounds described is given in Appendix C.

**Scheme 5:**
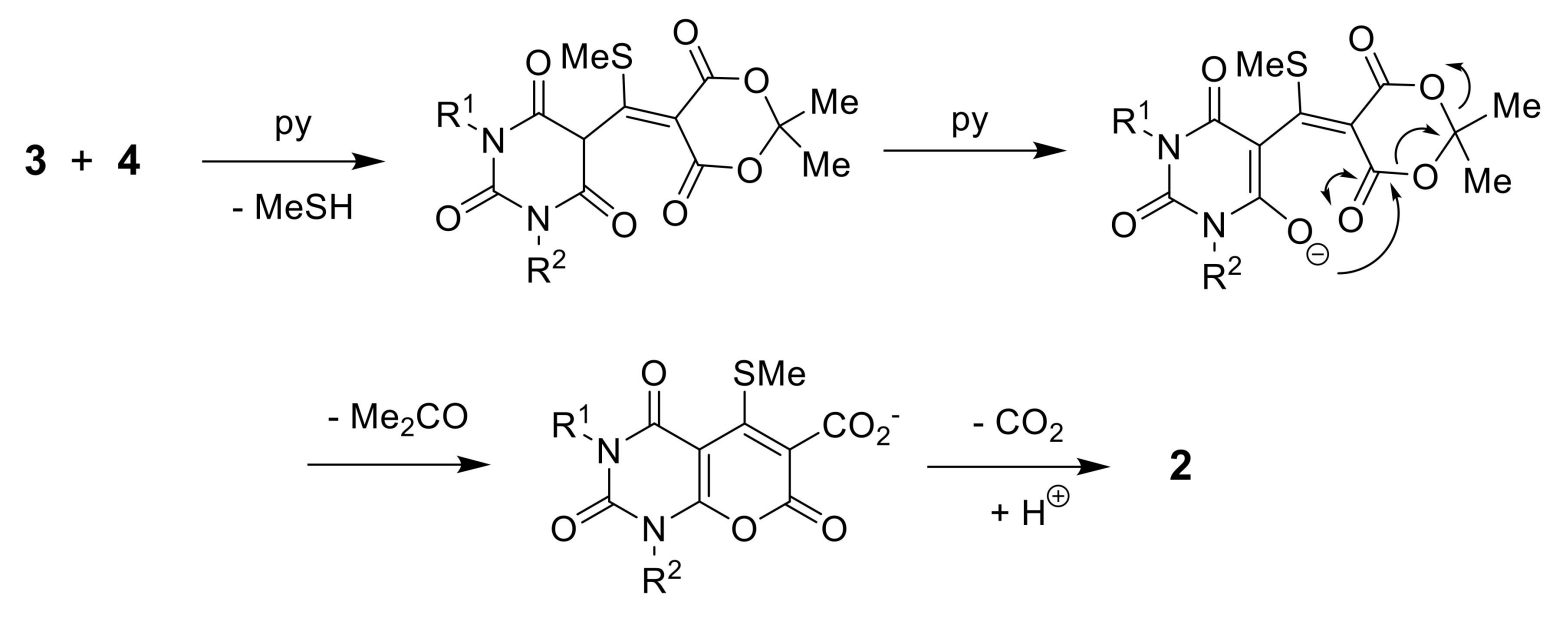
Pathway to compound **2** from the condensation of **3** with **4**.

For the reactions of **3** with **4,** where one possible regioisomer was formed preferentially, it is proposed that the carbanion derived from deprotonation of compound **3** at C-3 displaces one methylthio group to give an intermediate (Scheme 5), which can cyclise with loss of acetone, followed by CO_2_, to give **2a**–**d** or the regioisomer where R^1^ and R^2^ are interchanged. The preference for the regioisomer shown may be due to the reacting oxygen atom near to R^2^ (= H) being less sterically shielded than the corresponding oxygen near to R^1^ (= alkyl).

For efficient reactions of pyranopyrimidinones **9a**–**f** with amines to afford alkenes **10a**–**q** (Scheme 4), it was expedient to use TFE as a solvent with microwave irradiation. Previous studies have highlighted the advantages of TFE for reactions where its exceptional solvating and hydrogen bond donor properties are beneficial, as well as its suitability for microwave irradiation [15,16]. No reaction was observed when dimethylformamide was used as the solvent in attempts to convert compound **9** or its derivatives into **10**.

Biological evaluation of the compounds (see Table 1 and Figure A6, Appendix B) identified **9b**, **9c**, and **10b** had significant hits against *S. pombe*, with **9b** similar to the potency of nystatin. The *S. pombe* strain, developed for screening purposes, is a mutant strain with seven drug exporter genes deleted to make it hypersensitive to challenge with toxins [17,18]. It is interesting that the substituents R^1^, R^2,^ and R^3^ were the same for **8b**, **9b,** and **10a**, and for **8c**, **9c**, and **10b**. The difference between the compounds **8b/8c** and **10a/10b** is a methyl carboxylate in compounds **8b/8c** versus a morpholino-amide in **10a/10b**. Compounds **8b**/**8c**, **9b**/**9c**, and **10a**/**10b** differ solely by a methyl group in one versus a methoxy in the other. No compound where R^1^ = Et showed comparable activity to the best benzyl-substituted compounds. Comparing results for compounds **8**, **9,** and **10** indicates that substituents R^1^ and R^3^ were the primary drivers for potency. For compounds **10**, only when R^4^ contained a morpholino moiety was any activity observed. Further studies will be aimed at identifying the biological target for these compounds and extend structure-activity relationships by further exploring the parameters of group size, polarity, and hydrogen bonding capability. The ultimate aim is to identify lead compounds against pathogenic fungi.

## 4. Materials and Methods

### 4.1. General

All the materials used were purchased from Sigma-Aldrich (Merck Life Science UK Limited, Gillingham, Dorset, UK), Acros Organics (Fisher Scientific, Loughborough, Leicestershire, UK), or Fluorochem (Hadfield, Derbyshire, UK) and used without further purification. All reactions were monitored by thin-layer chromatography on 0.25 mm silica gel plates (60GF-254) and visualized with UV light. ^1^H and ^13^C-NMR spectra were recorded on a Bruker Avance300 spectrometer (Bruker UK Limited, Banner Lane Coventry CV4 9GH, UK) operating at 300.13 and 75.48 MHz, respectively, or a Bruker Avance400 spectrometer (Bruker UK Limited, Banner Lane Coventry CV4 9GH, UK) operating at 399.78 and 100.54 MHz, respectively, using TMS as an internal standard in DMSO-*d*_6_ or CDCl_3_ solutions. Chemical shifts were reported in delta (*δ*) units, parts per million (ppm) downfield from tetramethylsilane. LC-MS analyses were performed on a Shimadzu 2010EV (Shimadzu UK Limited, Buckinghamshire MK12 5RD, UK) instrument operating in negative (-ve) electrospray ionization (ESI) mode with UV detection at 254 nm.

Crystal structure data were collected at 150 K on a Rigaku Oxford Diffraction Xcalibur Atlas Gemini Ultra diffractometer equipped with a sealed tube X-ray source (λ_CuKα_ = 1.54184 Å) and an Oxford CryostreamPlus open-flow *N*_2_ cooling device. Intensities were corrected for absorption using a multifaceted crystal model created by indexing the faces of the crystal for which data were collected [19]. Cell refinement, data collection, and data reduction were undertaken via the software CrysAlisPro (Rigaku Oxford Diffraction, Tokyo, Japan).

All structures were solved using XT [20] and refined by XL [21] using the Olex2 interface [22]. All non-hydrogen atoms were refined as anisotropic, and hydrogen atoms were positioned with idealized geometry, with the exception of those bound to heteroatoms, the positions of which were located using peaks in the Fourier difference map. The displacement parameters of the hydrogen atoms were constrained using a riding model with *U*_H_ set to be an appropriate multiple of the *U*_eq_ value of the parent atom.

### 4.2. Synthesis

See Appendix A.

### 4.3. Biological Assays

The bacterial strains (*Escherichia coli* (DH5α), the Gram-positive bacterium *Staphylococcus aureus* (RN4220)) were grown in Luria Bertani (LB) medium (10 mL) overnight and then diluted 1:50 in fresh LB medium and grown for 6 h. *S pombe* was grown in YE6S medium overnight and then diluted 1:10 in fresh YE6S medium and grown for 6 h. *C albicans* was grown in Sabourand medium overnight and then diluted 1:10 in fresh Sabourand medium and grown for 6 h, then diluted 1:10 in fresh medium and the diluted culture was used in the assay.

For all organisms, 400 µL of culture was spotted onto a solid medium in a standard square plate and spread evenly over the surface by shaking with sterile glass beads. For the bacterial strains, Oxoid Nutrient Agar plates were used, whilst for *S. pombe* YE6S agar plates were used, and for *C. albicans*, Sabourand agar plates were used. Filter paper discs to which 5 µL of each test compound (10 mg/mL in DMSO, i.e., ~0.02 M for each compound) were placed on the plates, which were incubated overnight at 30 °C.

Control discs were used where 5 µL of the positive controls rifampicin (1 mg/mL in DMSO) and nystatin (50 mg/mL in DMSO, 0.054 M) were added.

After incubating overnight, the plates were inspected for a halo (zone of inhibition) surrounding the discs, and the plates were scanned. The *S. pombe* bioassay plates were incubated for a further 24 h and then reassessed and scanned. Owing to the slower growth of *S. pombe* (~3–4 h doubling time) compared to the bacterial strains (20–30 min doubling time) and the more vigorous *C. albicans*, a longer incubation period was required to produce a confluent lawn of *S. pombe* cells.

## 5. Conclusions

Routes are described herein affording a diverse library of tetrasubstituted alkenes 3-amino-3-(2,4,6-trioxotetrahydropyrimidin-5(2*H*)-ylidene)propenamides via pyranopyrimidinones (Scheme 1, Scheme 2, Scheme 3 and Scheme 4). Biological assays against selected bacterial and fungal organisms revealed significant activity for compounds **9b**, **9c,** and **10b** against the unicellular eukaryotic yeast *Schizosaccharomyces pombe*, with compound **9b** matching the potency of nystatin. This study has revealed novel structural motifs targeting the fungal organism *Schizosaccharomyces pombe* and provides the groundwork for identifying a lead against pathogenic fungi.

## Figures and Tables

**Table 1:** Selected bioassay results against *Schizosaccharomyces pombe*.

Compound	Average Zone of Inhibition (Radius, mm)
**5b**	1.5
**5c**	2
**8b**	1
**8c**	1.5
**8e**	0.5
**9b**	10
**9c**	5
**9e**	2.5
**9f**	3
**10a**	0.5
**10b**	5
Nystatin	8

## Appendix A. Crystallography

Crystals of compounds **2c**, **5c**, **7b**, **9c,** and **10b** were obtained by the recrystallization from dimethyl sulfoxide, ethanol, dichloromethane-diethyl ether (vapor diffusion), acetone-diethyl ether (vapor diffusion), and chloroform-diethyl ether (vapor diffusion), respectively. The structures were determined by single-crystal X-ray crystallography. CCDC deposition numbers are: 2026849 (**2c**), 2026850 (**5c**), 2026846 (**7b**), 2026847 (**9c**) and 2026848 (**10b**). Structures are shown in Appendix A, Figure A1, Figure A2, Figure A3, Figure A4 and Figure A5.

The crystal structure analysis of compound 5c confirmed its structure (Figure A2), showing that there was an intramolecular hydrogen bond between the amino NHR^3^ and the neighboring pyrimidine trione carbonyl group. The N···O (donor···acceptor) distance was 2.5661(16) Å, and the presence of the amino proton was confirmed by a prominent peak in the Fourier difference map. With the proton included, this intramolecular hydrogen bond formed a 6-membered S(6) ring motif with an H···O distance of 1.807(19) Å and an N-H···O angle of 144.0(17)°. The formation of this ring is expected according to Etter’s second rule of hydrogen bonds, which states that where an S(6)-type ring can form, it will, and that exceptions to this rule are rare [23]. The amide proton in this structure forms an intermolecular hydrogen bond to the amide carbonyl of an adjacent molecule. These interactions propagate along the crystallographic (001) direction to produce a C(4) hydrogen-bonded chain motif.

The structures of **7b**, **9c,** and **10b** are representative of the compound groups **7**, **9,** and **10**, respectively (Figure A3, Figure A4 and Figure A5). As observed in the structure of **5c**, an amino NH to carbonyl intramolecular hydrogen bond was also apparent in **9c** and **10b**, forming the S(6) ring motif. The proton of the barbituric acid moiety also forms a hydrogen bond in each of these structures, but unlike the structure of **5c**, where an amide proton formed the intermolecular bond, these interactions form closed dimers with each molecule related by inversion symmetry. In each case, the hydrogen-bonding motif was a ring, R22(8) in the structures of **7b** and **10b**, and a larger R22(12) ring for **9c**.

## Appendix B. Disk Assays

Figure A6 shows inhibition of the growth of *S. pombe* caused by compounds **5b**, **5c**, **8b**, **8c**, **8e**, **9b**, **9c**, **9e**, **9f**, **10a** and **10b** with nystatin as reference standard.

## Appendix C. Synthesis

### Appendix C.1. General Procedure A

*Pyrano[2,3-d]pyrimidinones***2a**–**e** Barbituric acid **3a**–**e** (4 mmol) and 5-[bis(methylthio)methylene]-2,2-dimethyl-1,3-dioxane-4,6-dione **4a**–**e** (4 mmol) were heated at reflux in pyridine for 1 h. The condenser was connected to a Dreschel bottle containing bleach solution to destroy methanethiol. The resulting precipitate was filtered off and washed with EtOH to give **2a**–**e**.

*3-Benzyl-5-(methylthio)-2H-pyrano[2,3-d]pyrimidine-2,4,7(1H,3H)-trione* (**2a**).

Yield: 60%; yellow solid, melting point 270–280 °C; ^1^H-NMR (300 MHz, DMSO-*d*_6_): 10.45 (s, 1H, NH), 7.30 (m, 5H, ArH), 5.68 (s, 1H, C=CH), 4.97 (s, 2H, ArCH_2_), 2.38 (s, 3H, SCH_3_); ^13^C-NMR (300 MHz, DMSO-*d*_6_): 168.8, 162.5, 155.6 and 153.3 (C=O), 149.2, 139.6, 137.2, 128.7, 127.9 and 127.4 (Ar), 96.6 (C=C), 43.6 (CH_2_), 15.2 (SCH_3_); LC-MS (ESI-ve) Found [M − H]^−^ 314.95 (C_15_H_11_N_2_O_4_S requires 315.04).

*3-Ethyl-5-(methylthio)-1H-pyrano[2,3-d]pyrimidine-2,4,7(3H)-trione* (**2b**). Yield: 69%; pale orange solid, melting point 301–303 °C; ^1^H-NMR (300 MHz, DMSO-*d*_6_): 13.10 (s, 1H, NH), 5.65 (s, 1H, C=CH), 3.79 (q, 2H, *J* = 7.0 Hz, C*H*_2_CH_3_), 2.38 (s, 3H, SCH_3_), 1.10 (t, 3H, *J* = 7.0 Hz, CH_2_C*H*_3_); ^13^C-NMR (300 MHz, DMSO-*d*_6_): 162.5, 160.1, 158.6 and 155.6 (C=O), 96.5 and 91.2 (C=C), 35.7 (*C*H_2_CH_3_), 15.2 (SCH_3_), 13.7 (CH_2_*C*H_3_); LC-MS (ESI-ve) Found [M − H]^−^ 252.95 (C_10_H_9_N_2_O_4_S requires 253.03).

*3-Methyl-5-(methylthio)-1H-pyrano[2,3-d]pyrimidine-2,4,7(3H)-trione* (**2c**).

Yield: 62%; yellow crystals, melting point 286–288 °C; ^1^H-NMR (300 MHz, DMSO-*d*_6_): 8.65 (s, 1H, NH), 5.62 (s, 1H, C=CH) 3.13 (s, 3H, NCH_3_), 2.38 (s, 3H, SCH_3_); ^13^C-NMR (300 MHz, DMSO-*d*_6_): 162.5, 160.4, 158.6 and 155.6 (C=O), 138.7, 125.1, 96.4 and 91.0 (C=C), 27.4 (NCH_3_), 15.2 (SCH_3_); LC-MS (ESI-ve) Found [M – H]^−^ 238.95 (C_9_H_7_N_2_O_4_S requires 239.01).

*3-(4-Fluorobenzyl)-5-(methylthio)-2H-pyrano[2,3-d]pyrimidine-2,4,7(1H,3H)-trione* (**2d**).

Yield: 61%; yellow-orange solid, melting point 204–206 °C; ^1^H-NMR (400 MHz, DMSO-*d*_6_): 8.66 (s, 1H, NH), 7.34-7.11 (m, 4H, ArH), 5.66 (s, 1H, C=CH) 4.94 (s, 2H, ArCH_2_), 2.37 (s, 3H, SCH_3_); ^13^C-NMR (400 MHz, DMSO-*d*_6_): 163.0, 162.5, 160.3 and 155.6 (C=O), 149.2, 133.4, 130.3 and 115.4 (Ar), 96.6 and 91.3 (C=C), 43.0 (CH_2_), 15.2 (SCH_3_); LC-MS (ESI-ve) Found [M − H]^−^ 332.90 (C_15_H_10_FN_2_O_4_S requires 333.03).

*1,3-Dimethyl-5-(methylthio)-1H-pyrano[2,3-d]pyrimidine-2,4,7(3H)-trione* (**2e**).

Yield: 67%; yellow crystals, melting point 275–278 °C; ^1^H-NMR (400 MHz, DMSO-*d*_6_): 5.56 (s, 1H,C=CH), 3.26, 3.46 (2s, 6H, NCH_3_), 2.31 (s, 3H, SCH_3_); ^13^C-NMR (400 MHz, DMSO-*d*_6_): 164.0 (C=O), 96.5 (C=C), and 29.8 28.5 (NCH_3_), 16.0 (SCH_3_). LC-MS (ESI + ve) Found [M + H]^+^ 254.90 (C_1__0_H_1__1_N_2_O_4_S requires 255.04).

### Appendix C.2. General Procedure B

*3-Amino-3-(2,4,6-trioxotetrahydropyrimidin-5(2H)-ylidene)propanamides***5a**–**d**. A solution of 1,3-dimethyl-5-(methylthio)-1H-pyrano[2,3-*d*]pyrimidine-2,4,7(3H)-trione **2e** (2 mmol) and benzylamine (4 mmol) was heated at reflux in EtOH (10 mL) for 1 h. The EtOH was removed, and the residue was washed with diethyl ether to give **5**(**a**–**d**).

*N-benzyl-3-(benzylamino)-3-(1,3-dimethyl-2,4,6-trioxotetrahydropyrimidin-5(2H)-ylidene)propenamide* (**5a**).

Yield: 79%; white crystals, melting point 186–188 °C; ^1^H-NMR (400 MHz, CDCl_3_): 13.02 (s, 1H, NH), 7.93 (s, 1H, NH), 7.33–7.22 (m, 10H, ArH), 5.04 (s, 2H, NCH_2_Ar), 4.36 (s, 2H, CONCH_2_Ar), 4.17 (s, 2H, COCH_2_), 3.24 and 3.20 (s, 6H, NCH_3_); ^13^C-NMR (400 MHz, CDCl_3_): 169.0, 166.6, 166.3 and 164.0 (C=O), 151.0, 137.9, 135.4, 129.1, 128.6, 128.2, 127.4 and 127.3 (Ar), 90.5 (C=C), 48.2, 43.7 and 37.8 (CH_2_), 28.2 and 27.8 (CH_3_); LC-MS (ESI-ve) Found [M − H]^−^ 419.15 (C_23_H_23_N_4_O_4_ requires 419.17).

*N-(4-chloro-3-(trifluoromethyl)benzyl)-3-((4-chloro-3-(trifluoromethyl)benzyl)amino)-3-(1,3-dimethyl-2,4,6-trioxotetrahydropyrimidin-5(2H)-ylidene)propenamide* (**5b**).

Yield: 78%; white crystals, melting point 209–211 °C; ^1^H-NMR (400 MHz, CDCl_3_): 13.08 (s, 1H, NH), 8.16 (s, 1H, NH), 7.58–7.31 (m, 6H, ArH), 5.07 (s, 2H, NCH_2_Ar), 4.37 (s, 2H, CONCH_2_Ar), 4.14 (s, 2H, COCH_2_), 3.26 and 3.24 (s, 6H, NCH_3_); ^13^C-NMR (400 MHz, CDCl_3_): 168.7, 166.8, 166.4 and 164.0 (C=O), 150.8, 137.2, 134.6, 132.3, 131.8, 131.7, 126.8, 126.7, 126.2 and 126.1 (Ar), 96.2 (C=C), 47.2, 42.7 and 37.7 (CH_2_), 28.3 and 27.9 (CH_3_); LC-MS (ESI-ve) Found [M − H]^−^ 623.00 (C_25_H_19_^35^Cl_2_F_6_N_4_O_4_ requires 623.07).

*3-(1,3-Dimethyl-2,4,6-trioxotetrahydropyrimidin-5(2H)-ylidene)-N-(3-(trifluoromethyl)benzyl)-3-((3-(trifluoromethyl)benzyl)amino)propenamide* (**5c**).

Yield: 55%; white crystals, melting point 148–149 °C; ^1^H-NMR (400 MHz, CDCl_3_): 13.10 (s, 1H, NH), 8.11 (s, 1H, NH), 7.54–7.38 (m, 8H, ArH), 5.11 (s, 2H, NCH_2_Ar), 4.43 (s, 2H, CONCH_2_Ar), 4.18 (s, 2H, COCH_2_), 3.26 and 3.23 (s, 6H, NCH_3_); ^13^C-NMR (400 MHz, CDCl_3_): 168.9, 166.7, 166.4 and 164.1 (C=O), 150.8, 139.0, 136.5, 130.6, 129.8, 129.1, 125.2, 124.3, 124.2 and 123.8 (Ar), 90.8 (C=C), 47.7, 43.1 and 37.7 (CH_2_), 28.2 and 27.9 (CH_3_); LC-MS (ESI-ve) Found [M − H]^−^ 555.05 (C_25_H_21_F_6_N_4_O_4_ requires 555.15), HRMS (ESI + ve) Found [M + H]^+^ 557.1234 (C_25_H_23_F_6_N_4_O_4_ requires 557.1623).

*N-(4-chlorobenzyl)-3-((4-chlorobenzyl)amino)-3-(1,3-dimethyl-2,4,6-trioxotetrahydropyrimidin-5(2H)-ylidene)propenamide* (**5d**).

Yield: 80%; white crystals, melting point 216–217 °C; ^1^H-NMR (400 MHz, CDCl_3_): 12.99 (s, 1H, NH), 8.00 (s, 1H, NH), 7.28–7.10 (m, 8H, ArH), 5.00 (s, 2H, NCH_2_Ar), 4.31 (s, 2H, CONCH_2_Ar), 4.13 (s, 2H, COCH_2_), 3.25 and 3.22 (s, 6H, NCH_3_); ^13^C-NMR (400 MHz, CDCl_3_): 168.8, 166.6, 166.3 and 164.0 (C=O), 150.9, 136.4, 134.3, 133.8, 133.2, 129.4, 128.9, 128.8 and 128.7 (Ar), 90.7 (C=C), 47.6, 43.1 and 37.8 (CH_2_), 28.3 and 27.9 (CH_3_); LC-MS (ESI-ve) Found [M − H]^−^ 487.10 (C_23_H_21_Cl_2_N_4_O_4_ requires 487.09).

*(E/Z)-3-(1-(4-fluorobenzyl)-2,4,6-trioxotetrahydropyrimidin-5(2H)-ylidene)-N-(4-methoxyphenyl)-3-((4-methoxyphenyl)amino)propanamide***5e**.

A solution of 3-(4-fluorobenzyl)-5-(methylthio)-2H-pyrano[2,3-d]pyrimidine-2,4,7(1H,3H)-trione **2d** (1 mmol) and 4-methoxyaniline (1 mmol) was heated at 150 °C in DMSO for 2 h. After cooling to RT, the resulting precipitate was filtered off and washed with diethyl ether to give **5e**.

Yield: 82%; white solid, no clear melting point (decomposed); ^1^H-NMR (300 MHz, DMSO-*d*_6_): 13.99 and 13.72 (s, 1H, NH, *E,Z*), 11.33 and 10.96 (s, 1H, NH, *E,Z*), 9.93 and 9.91 (s, 1H, NH, *E,Z*), 7.39–6.85 (m, 12H, ArH), 4.98 and 4.90 (s, 2H, ArCH_2_, *E,Z*), 4.04 and 4.01 (s, 2H, COCH_2_, *E,Z*), 3.77 and 3.72 (s, 6H, 2 × OCH_3_); ^13^C-NMR (300 MHz, DMSO-*d*_6_): 170.1, 169.5, 167.2, 165.7, 163.1 and 162.8 (C=O), 159.4, 155.5, 150.3, 132.6, 130.1, 130.0, 129.8, 129.7, 128.8, 127.6, 121.0, 120.9, 115.7, 115.5, 115.4, 115.2 and 114.3 (Ar), 91.0 (C=C), 55.9 and 55.6 (OCH_3_), 42.3 (CH_2_); LC-MS (ESI-ve) Found [M − H]^−^ 531.15 (C_28_H_24_FN_4_O_6_ requires 531.17).

### Appendix C.3.General Procedure C

*3-Amino-3-(2,4,6-trioxotetrahydropyrimidin-5(2H)-ylidene)propanamides***5f**–**l**. A solution of 1,3-dimethyl-5-(methylsulfinyl)-2*H*-pyrano[2,3-*d*]pyrimidine-2,4,7(1*H*,3*H*)-trione **6** (2 mmol) and aniline (4 mmol) was heated at reflux in EtOH (10 mL) for 1 h. The ethanol was removed and the residue was washed with diethyl ether to give **5f**–**l**.

3-((4-Chloro-3-(trifluoromethyl)phenyl)amino)-N-(3-chloro-4-(trifluoromethyl)phenyl)-3-(1,3-dimethyl-2,4,6-trioxotetrahydropyrimidin-5(2H)-ylidene)propenamide (**5f**).

Yield: 61%; white crystals, melting point 212–215 °C; ^1^H-NMR (300 MHz, DMSO-*d*_6_): 14.02 (s, 1H, NH), 10.61 (s, 1H, NH), 8.12-7.63 (m, 6H, ArH), 4.12 (s, 2H, COCH_2_), 3.24 and 3.14 (s, 6H, NCH_3_); ^13^C-NMR (400 MHz, CDCl_3_): 168.7, 167.2, 166.2 and 162.4 (C=O), 150.9, 138.8, 136.0, 133.4, 132.6, 132.3, 126.4, 124.1 and 117.8 (Ar), 95.9 and 91.8 (C=C), 28.2 and 28.0 (CH_3_); LC-MS (ESI-ve) Found [M − H]^−^ 594.95 (C_23_H_1__5_^35^Cl_2_F_6_N_4_O_4_ requires 595.04).

*N-3-((trifluoromethyl)phenyl)-3-(1,3-dimethyl-2,4,6-trioxotetrahydropyrimidin-5(2H)-ylidene)-3-((3-(trifluoromethyl)phenyl)amino)propenamide* (**5g**).

Yield: 72%; white crystals, melting point 215–218 °C; ^1^H-NMR (400 MHz, CDCl_3_): 14.53 (s, 1H, NH), 8.96 (s, 1H, NH), 7.74–7.26 (m, 8H, ArH), 4.06 (s, 2H, COCH_2_), 3.31 and 3.27 (s, 6H, NCH_3_); ^13^C-NMR (400 MHz, CDCl_3_): 167.9, 166.6, 164.9 and 163.8 (C=O), 150.7, 138.2, 136.4, 130.4, 129.5, 125.5, 123.6, 122.7, 121.0 and 116.5 (Ar), 91.8 (C=C), 39.8 (CH_2_), 28.4 and 28.0 (CH_3_); LC-MS (ESI-ve) Found [M − H]^−^ 527.00 (C_23_H_1__7_F_6_N_4_O_4_ requires 527.12).

*N-(4-chlorophenyl)-3-((4-chlorophenyl)amino)-3-(1,3-dimethyl-2,4,6-trioxotetrahydropyrimidin-5(2H)-ylidene)propenamide* (**5h**).

Yield: 60%; white solid, melting point 273–276 °C; ^1^H-NMR (400 MHz, CDCl_3_): 14.36 (s, 1H, NH), 8.79 (s, 1H, NH), 7.39-7.15 (m, 8H, ArH), 4.06 (s, 2H, COCH_2_), 3.30 and 3.26 (s, 6H, 2 × NCH_3_); ^13^C-NMR (400 MHz, CDCl_3_): 168.1, 166.6, 164.8 and 163.7 (C=O), 150.8, 136.3, 134.8, 134.2, 129.8, 128.9, 128.0 and 121.0 (Ar), 91.5 (C=C), 39.7 (CH_2_), 28.3 and 28.0 (CH_3_); LC-MS (-ve) Found [M − H]^−^ 459.05 (C_21_H_1__7_^35^Cl_2_N_4_O_4_ requires 459.06).

*3-(1,3-Dimethyl-2,4,6-trioxotetrahydropyrimidin-5(2H)-ylidene)-N-(4-phenoxyphenyl)-3-((4-phenoxyphenyl)amino)propenamide* (**5i**).

Yield: 75%; white crystals, melting point 207–210 °C; ^1^H-NMR (400 MHz, CDCl_3_): 14.28 (s, 1H, NH), 8.63 (s, 1H, NH), 7.37–6.98 (m, 18H, ArH), 4.12 (s, 2H, COCH_2_), 3.30 and 3.27 (s, 6H, 2 × NCH_3_); ^13^C-NMR (400 MHz, CDCl_3_): 168.5, 166.6, 164.9 and 163.7 (C=O), 157.9, 156.0, 153.5, 150.9, 133.4, 129.7, 128.1, 124.3, 123.0, 121.5, 119.7 and 118.8 (Ar), 96.2 and 91.0 (C=C), 39.6 (CH_2_), 28.3 and 27.9 (NCH_3_); LC-MS (ESI-ve) Found [M − H]^−^ 575.10 (C_33_H_27_N_4_O_6_ requires 575.19).

*3-(1,3-Dimethyl-2,4,6-trioxotetrahydropyrimidin-5(2H)-ylidene)-N-(4-methoxyphenyl)-3-((4-methoxyphenyl)amino)propenamide* (**5j**).

Yield: 77%; white solid, no clear melting point (decomposed); ^1^H-NMR (400 MHz, CDCl_3_): 14.19 (s, 1H, NH), 8.50 (s, 1H, NH), 7.28–6.71 (m, 8H, ArH), 4.07 (s, 2H, COCH_2_), 3.76 and 3.69 (s, 6H, 2 × OCH_3_), 3.29 and 3.25 (s, 6H, NCH_3_); ^13^C-NMR (400 MHz, CDCl_3_): 168.7, 166.5, 164.7 and 163.6 (C=O), 159.6, 156.4, 151.0, 131.1, 128.4, 127.8, 121.5, 114.7 and 114.0 (Ar), 96.2 and 91.0 (C=C), 55.5 (OCH_3_), 39.6 (CH_2_), 28.2 and 27.9 (NCH_3_); LC-MS (ESI-ve) Found [M − H]^−^ 451.15 (C_23_H_23_N_4_O_6_ requires 451.16).

*3-(1,3-Dimethyl-2,4,6-trioxotetrahydropyrimidin-5(2H)-ylidene)-N-(p-tolyl)-3-(p-tolylamino)propenamide* (**5k**).

Yield: 64%; white crystals, melting point decomposed; ^1^H-NMR (400 MHz, CDCl_3_): 14.26 (s, 1H, NH), 8.49 (s, 1H, NH), 7.27–6.98 (m, 8H, ArH), 4.08 (s, 2H, COCH_2_), 3.29 and 3.25 (s, 6H, 2 × NCH_3_), 2.31 and 2.21 (s, 6H, ArCH_3_); ^13^C-NMR (400 MHz, CDCl_3_): 168.5, 166.6, 164.8 and 163.6 (C=O), 151.0, 138.7, 135.3 133.9, 133.2, 130.2, 129.4, 126.3 and 119.8 (Ar), 96.2 and 91.1 (C=C), 39.7 (CH_2_), 28.2 and 27.9 (NCH_3_), 21.1 and 20.9 (ArCH_3_); LC-MS (ESI-ve) Found [M − H]^−^ 419.15 (C_23_H_23_N_4_O_4_ requires 419.17).

*3-(1,3-Dimethyl-2,4,6-trioxotetrahydropyrimidin-5(2H)-ylidene)-N-(4-fluorophenyl)-3-((4-fluorophenyl)amino)propenamide* (**5l**).

Yield: 80%; white solid, melting point 249-252 °C; ^1^H-NMR (300 MHz, CDCl_3_): 14.303 (s, 1H, NH), 8.69 (s, 1H, NH), 7.33–6.90 (m, 8H, ArH), 4.06 (s, 2H, COCH_2_), 3.30 and 3.27 (s, 6H, 2 × NCH_3_); ^13^C-NMR (300 MHz, CDCl_3_): 168.4, 166.6, 164.0 and 163.6 (C=O), 150.8, 128.6, 121.6, 116.8, 116.4, 115.7 and 115.5 (Ar), 39.5 (CH_2_), 28.3 and 27.9 (CH_3_); LC-MS (ESI-ve) Found [M − H]^−^ 427.05 (C_21_H_1__7_F_2_N_4_O_4_ requires 427.12).

*3-Dimethyl-5-(methylsulfinyl)-2H-pyrano[2,3-d]pyrimidine-2,4,7(1H,3H)-trione***6**. To a solution of **2e** (2.6 g, 10 mmol) in DCM (20 mL), *m*-chloroperbenzoic acid (1.73 g, 10 mmol) was added at RT. The mixture was stirred for 2 h. The DCM was removed, and the residual solid was briefly stirred in diethyl ether (20 mL). The mixture was filtered, and the solid collected was dried under a vacuum. The crude product was recrystallized from DCM-Et_2_O.

Yield: 58%; pale yellow crystals, melting point 188–190 °C; ^1^H-NMR (400 MHz, DMSO-*d*_6_): 6.66 (s, 1H, C=CH), 3.31, 3.53 (2s, 6H, 2 × NCH_3_), 2.78 (s, 3H, S(O)CH_3_); ^13^C-NMR (400 MHz, DMSO-*d*_6_): 167.6 and 154.9 (C=O), 104.0 (C=C), 44.8 (S(O)CH_3_), 30.2 and 31.6 (NCH_3_). LC-MS (ESI + ve) Found [M + H]^+^ 270.90 (C_10_H_11_N_2_O_5_S requires 271.03).

### Appendix C.4.General Procedure D

*(E/Z)-3-(2,4,6-trioxotetrahydropyrimidin-5(2H)-ylidene)-3-(methylthio)propanoates***7a**–**b**. To a solution of pyrano[2,3-*d*]pyrimidine **2a**–**b** (2 mmol) in MeOH (5 mL) was added sodium methoxide (2 mmol, 25% solution in MeOH), and the resulting solution was heated at reflux overnight. The MeOH was removed, and H_2_O (25 mL) was added. The solution was neutralized with aq. 1 M HCl. The resulting precipitate was filtered off and washed with water to give **7****a**–**b**.

*(E/Z)-3-(1-benzyl-2,4,6-trioxotetrahydropyrimidin-5(2H)-ylidene)-3-(methylthio)propanoate* (**7a**).

Yield: 71%; pale yellow solid, melting point 172–175 °C; ^1^H-NMR (400 MHz, CDCl_3_): 8.75 and 8.67 (s, 1H, NH, *E,Z*), 7.38–7.19 (m, 5H, ArH), 5.00 and 5.01 (s, 2H, ArCH_2_, *E,Z*), 4.430 and 4.40 (s, 2H, COCH_2_, *E,Z*), 3.68 and 3.65 (s, 3H, OCH_3_), 2.38 (s, 3H, SCH_3_); ^13^C-NMR (400 MHz, CDCl_3_): 182.8, 167.8, 162.9 and 160.2 (C=O), 149.4, 136.5, 129.2, 129.0, 128.6, 128.4, 128.1 and 127.6 (Ar), 112.7 (*C*=CSCH_3_), 60.0 (C=*C*SCH_3_), 52.8 (OCH_3_), 44.1 (ArCH_2_), 39.6 (COCH_2_), 17.1 (SCH_3_); LC-MS (ESI-ve) Found [M − H]^−^ 347.00 (C_16_H_1__5_N_2_O_5_S requires 347.07), HRMS (ESI + ve) Found [M + Na]^+^ 371.07 (C_16_H_1__6_N_2_O_5_SNa requires 371.07).

*(E/Z)-3-(1-ethyl-2,4,6-trioxotetrahydropyrimidin-5(2H)-ylidene)-3-(methylthio)propanoate* (**7b**).

Yield: 77%; orange solid, melting point 189–191 °C; ^1^H-NMR (300 MHz, CDCl_3_): 8.55 and 8.37 (s, 1H, NH, *E,Z*), 4.47 and 4.30 (s, 2H, COCH_2_), 3.90 and 3.78 (q, *J* = 7.1 Hz, 2H, CH_3_C*H*_2_, *E,Z*), 3.71 (s, 3H, OCH_3_), 2.41 (s, 3H, SCH_3_), 1.28 and 1.17 (t, *J* = 7.0 Hz, 3H, C*H*_3_CH_2_); ^13^C-NMR (300 MHz, CDCl_3_): 182.3, 167.9, 160.1 and 160.1 (C=O), 52.8 (COOCH_3_), 39.5 (COCH_2_), 36.3 (*C*H_2_CH_3_), 17.1 (SCH_3_), 13.4 (CH_2_*C*H_3_); LC-MS (ESI-ve) Found [M − H]^−^ 284.90 (C_11_H_1__3_N_2_O_5_S requires 285.05).

### Appendix C.5. General Procedure E

*Methyl (E/Z)-(2,4,6-trioxotetrahydropyrimidin-5(2H)-ylidene)-3-(amino)propanoates***8a**–**f**. A solution of (*E,Z*)-3-(2,4,6-trioxotetrahydropyrimidin-5(2H)-ylidene)-3-(methylthio)propanoate **7a**–**b** (1 mmol) and amine (2 mmol) in EtOH (15 mL) was heated at reflux overnight. After cooling to RT, the precipitate was filtered to give **8a**–**f**.

*Methyl (E/Z)-3-(1-benzyl-2,4,6-trioxotetrahydropyrimidin-5(2H)-ylidene)-3-(phenylamino)propanoate* (**8a**).

Yield: 61%; off-white solid, melting point 162–165 °C; ^1^H-NMR (300 MHz, CDCl_3_): 13.93 and 13.82 (s, 1H, NH, *E,Z*), 8.52 and 8.40 (s, 1H, NH, *E,Z*), 7.25–7.12 (m, 10H, ArH), 5.03 and 5.00 (s, 2H, ArCH_2_, *E,Z*), 3.90 and 3.80 (s, 2H, COCH_2_, *E,Z*), 3.66 and 3.63 (s, 3H, OMe); ^13^C-NMR (300 MHz, CDCl_3_): 168.2, 167.6, 166.3 and 162.7 (C=O), 150.0, 136.8, 135.5, 129.9, 128.8, 128,.3, 127.6, 125.9 (Ar), 91.2 (C=C), 52.6(OCH_3_), 43.7 (ArCH_2_), 38.0 (COCH_2_). LC-MS (ESI-ve) Found [M − H]^−^ 392.05 (C_21_H_1__8_N_3_O_5_ requires 392.12).

*Methyl (E/Z)-3-(1-benzyl-2,4,6-trioxotetrahydropyrimidin-5(2H)-ylidene)-3-((4-methoxyphenyl)amino) propanoate* (**8b**).

Yield: 82%; off-white solid, melting point 189–191 °C; ^1^H-NMR (300 MHz, CDCl_3_): 13.75 and 13.62 (s, 1H, NH, *E,Z*), 8.13 and 8.03 (s, 1H, NH, *E,Z*), 7.39–6.84 (m, 9H, ArH), 5.03 and 5.00 (s, 2H, ArCH_2_, *E,Z*), 3.89 and 3.86 (s, 2H, COCH_2_, *E,Z*), 3.76 and 3.67 (s, 3H, ArOCH_3_) 3.66 and 3.63 (s, 3H, OCH_3_); ^13^C-NMR (300 MHz, CDCl_3_): 168.2, 167.6, 166.3 and 162.7 (C=O), 150.0, 128.5, 128.3, 127.6, 127.2, 127.1, 115.0 and 114.7 (Ar), 91.2 (C=C), 55.6 (ArOCH_3_), 52.6 (COOCH_3_), 43.7 (ArCH_2_), 37.9 (COCH_2_); LC-MS (ESI-ve) Found [M − H]^−^ 422.10 (C_22_H_20_N_3_O_6_ requires 422.13).

*Methyl (E/Z)-3-(1-benzyl-2,4,6-trioxotetrahydropyrimidin-5(2H)-ylidene)-3-((p-tolylphenyl)amino)-propanoate* (**8c**).

Yield: 57%; orange crystals, melting point 171–173 °C; ^1^H-NMR (300 MHz, CDCl_3_): 13.93 and 13.82 (s, 1H, NH, *E,Z*), 8.53 and 8.43 (s, 1H, NH, *E,Z*), 7.49–7.12 (m, 9H, ArH), 5.13 and 5.10 (s, 2H, ArCH_2_, *E,Z*), 3.97 and 3.96 (s, 2H, COCH_2_, *E,Z*), 3.76 and 3.72 (s, 3H, ArOCH_3_) 2.4 (s, 3H, ArCH_3_); ^13^C-NMR (300 MHz, CDCl_3_): 168.5, 167.6, 166.4, 162.8 (C=O), 151.1, 130.4, 128.5, 127.6 and 125.7 (Ar), 52.5 (COOCH_3_), 43.7 (ArCH_2_), 37.9 (COCH_2_), 21.1 (ArCH_3_); LC-MS (ESI-ve) Found [M − H]^−^ 406.10 (C_22_H_20_N_3_O_5_ requires 406.14).

*Methyl (E,Z)-3-(1-ethyl-2,4,6-trioxotetrahydropyrimidin-5(2H)-ylidene)-3-((4-methoxyphenyl)amino) propanoate* (**8d**)**.**

Yield: 53%; off-white crystals, melting point 199–202 °C; ^1^H-NMR (300 MHz, CDCl_3_): 13.86 and 13.67 (s, 1H, NH, *E,Z*), 8.47 and 8.30 (s, 1H, NH, *E,Z*), 7.06–6.86 (m, 4H, ArH), 3.93 and 3.92 (q, *J* = 7.1 Hz, 2H, CH_3_C*H*_2_, *E,Z*), 3.89 and 3.88 (s, 2H, COCH_2_), 3.77 and 3.69 (s, 3H, ArOCH_3_), 1.20 and 1.11 (t, *J* = 7.1 Hz, 3H, C*H*_3_CH_2_); ^13^C-NMR (300 MHz, CDCl_3_): 168.6, 166.3, 162.7 and 159.6 (C=O), 149.7, 128.1, 127.3, 127.2 and 115.0 (Ar), 91.1 (C=C), 55.6 (ArOCH_3_), 52.6 (COOCH_3_), 37.9 (COCH_2_), 35.8 (*C*H_2_CH_3_), 13.4 (CH_2_*C*H_3_); LC-MS (ESI-ve) Found [M − H]^−^ 360.10 (C_17_H_1__8_N_3_O_6_ requires 360.12).

*Methyl (E/Z)-3-(1-ethyl-2,4,6-trioxotetrahydropyrimidin-5(2H)-ylidene)-3-((p-tolylphenyl)amino)-propanoate* (**8e**).

Yield: 82%; pale yellow solid, melting point 191–192 °C; ^1^H-NMR (300 MHz, CDCl_3_): 14.03 and 13.84 (s, 1H, NH, *E,Z*), 8.68 and 8.50 (s, 1H, NH, *E,Z*), 7.28–7.11 (m, 4H, ArH), 4.01 and 3.98 (q, *J* = 7.1 Hz, 2H, CH_3_C*H*_2_, *E,Z*), 3.94 and 3.93 (s, 2H, COCH_2_), 2.40 (s, 3H, ArCH_3_), 1.29 and 1.18 (t, *J* = 7.1 Hz, 3H, C*H*_3_-CH_2_); ^13^C-NMR (300 MHz, CDCl_3_): 168.5, 167.6, 166.4 and 162.8 (C=O), 149.7, 138.9, 133.0, 130.4, 125.8 and 125.7 (Ar), 91.2 (C=C), 52.5 (COOCH_3_), 37.9 (COCH_2_), 35.8 (*C*H_2_CH_3_), 21.1 (ArCH_3_), 13.4 (CH_2_*C*H_3_); LC-MS (ESI-ve) Found [M − H]^−^ 344.05 (C_17_H_1__8_N_3_O_5_ requires 344.12).

*Methyl (E/Z)-3-(1-ethyl-2,4,6-trioxotetrahydropyrimidin-5(2H)-ylidene)-3-((4-(trifluoromethyl)phenyl) amino)propanoate* (**8f**).

Yield: 56%; white crystals, melting point 193–194 °C; ^1^H-NMR (300 MHz, CDCl_3_): 14.03 and 13.84 (s, 1H, NH, *E,Z*), 8.68 and 8.50 (s, 1H, NH, *E,Z*), 7.28–7.11 (m, 4H, ArH), 4.01 and 3.98 (q, *J* = 7.1 Hz, 2H, CH_3_C*H*_2_, *E,Z*), 3.94 and 3.93 (s, 2H, COCH_2_), 1.29 and 1.18 (t, *J* = 7.1 Hz, 3H, C*H*_3_CH_2_); LC-MS (ESI-ve) Found [M − H]^−^ 398.00 (C_17_H_1__5_F_3_N_3_O_5_ requires 398.10).

### Appendix C.6. General Procedure F

*5-(Amino)-2H-pyrano[2,3-d]pyrimidine-2,4,7(1H,3H)-triones***9a**–**f**. To a stirred solution of methyl(*E*, *Z*)-(2,4,6-trioxotetrahydropyrimidin-5(2H)-ylidene)-3-(amino)propanoate **8a**–**f** (1 mmol) in MeOH (10 mL) was added aq. 1 M NaOH (1 mL). The mixture was heated to 70 °C for 1 h. The MeOH was removed, and H_2_O (20 mL) was added. The mixture was cooled in an ice bath and acidified with aq. 1 M HCl. The precipitate was filtered off and dried to give **9a**–**f**.

*3-Benzyl-5-(phenylamino)-2H-pyrano[2,3-d]pyrimidine-2,4,7(1H,3H)-trione* (**9a**).

Yield: 58%; white solid, no clear melting point (decomposed); ^1^H-NMR (300 MHz, DMSO-*d*_6_): 10.41 (s, 1H, NH, *E,Z*), 7.49–7.28 (m, 10H, ArH), 5.03 (s, 2H, ArCH_2_, *E,Z*), 4.91 (s, 1H, C=CH); ^13^C-NMR (300 MHz, DMSO-*d*_6_): 163.2, 160.9, 158.2 and 155.6 (C=O), 149.1, 137.3, 137.0, 130.1, 128.8, 127.8, 126.8 and 125.0 (Ar), 75.6 (C=C), 43.2 (ArCH_2_); LC-MS (ESI-ve) Found [M − H]^−^ 360.10 (C_20_H_1__4_N_3_O_4_ requires 360.10).

*3-Benzyl-5-((4-methoxyphenyl)amino)-2H-pyrano[2,3-d]pyrimidine-2,4,7(1H,3H)-trione* (**9b**).

Yield: 79%; white crystals, no clear melting point (decomposed); ^1^H-NMR (300 MHz, DMSO-*d*_6_): 10.18 (s, 1H, NH), 7.33–6.99 (m, 9H, ArH), 5.02 (s, 2H, ArCH_2_), 4.70 (s, 1H, C=CH); ^13^C-NMR (300 MHz, DMSO-*d*_6_): 163.2, 161.1, 158.3 and 156.5 (C=O), 149.4, 137.1, 129.9, 128.8, 127.8, 127.6, 127.0 and 115.1 (Ar), 84.4 and 75.0 (C=C), 55.8 (ArOCH_3_), 43.6 (ArCH_2_); LC-MS (ESI-ve) Found [M-H]^−^ 390.05 (C_21_H_1__6_N_3_O_5_ requires 390.11), HRMS (ESI + ve) Found [M + H]^+^ 392.09 (C_21_H_1__8_N_3_O_5_ requires 392.12).

*3-Benzyl-5-((p-tolylphenyl)amino)-2H-pyrano[2,3-d]pyrimidine-2,4,7(1H,3H)-trione* (**9c**).

Yield: 75%; white solid, no clear melting point (decomposed); ^1^H-NMR (300 MHz, DMSO-*d*_6_): 10.31 (s, 1H, NH), 7.34–7.22 (m, 9H, ArH), 5.02 (s, 2H, ArCH_2_), 4.84 (s, 1H, C=CH), 2.33 (s, 3H, ArCH_3_); ^13^C-NMR (300 MHz, DMSO-*d*_6_): 163.1, 160.8, 158.1 and 155.8 (C=O), 149.0, 137.0, 136.3, 134.6, 130.5, 128.8, 127.8 and 125.0 (Ar), 75.3 (C=C), 43.6 (ArCH_2_), 21.0 (ArCH_3_); LC-MS (ESI-ve) Found [M-H]^−^ 374.10 (C_21_H_1__6_N_3_O_4_ requires 374.11), HRMS (ESI + ve) Found [M + H]^+^ 376.10 (C_21_H_1__8_N_3_O_4_ requires 376.13).

*3-Ethyl-5-((4-methoxyphenyl)amino)-2H-pyrano[2,3-d]pyrimidine-2,4,7(1H,3H)-trione* (**9d**).

Yield: 53%; pale grey solid, no clear melting point (decomposed); ^1^H-NMR (300 MHz, DMSO-*d*_6_): 10.79 (s, 1H, NH), 7.26–6.93 (m, 4H, ArH), 4.57 (s, 1H, C=CH), 3.84 (q, *J* = 7.0 Hz, 2H, *CH*_2_CH_3_), 3.77 (s, 3H, ArOCH_3_), 1.05 (t, *J* = 7.0 Hz, 3H, CH_2_*CH*_3_); ^13^C-NMR (300 MHz, DMSO-*d*_6_): 167.8, 165.2, 162.1 and 157.6 (C=O), 131.3, 126.3 and 115.0 (Ar), 82.6 and 75.3 (C=C), 55.7 (ArOCH_3_), 34.8 (*C*H_2_CH_3_), 13.7 (CH_2_*C*H_3_); LC-MS (ESI-ve) Found [M − H]^−^ 328.05 (C_16_H_1__4_N_3_O_5_ requires 328.09).

*3-Ethyl-5-((p-tolylphenyl)amino)-2H-pyrano[2,3-d]pyrimidine-2,4,7-(1H,3H)-trione* (**9e**).

Yield: 46%; yellow-orange crystals, no clear melting point (decomposed); ^1^H-NMR (400 MHz, DMSO-*d*_6_): 13.10 (s, 1H, NH), 10.40 (s, 1H, NH), 7.26 (m, 4H, ArH), 4.84 (s, 1H, C=CH), 3.86 (q, *J* = 7.0 Hz, 2H, *CH*_2_CH_3_), 2.32 (s, 3H, ArCH_3_), 1.15 (t, *J* = 7.0 Hz, 3H, J = 7.0 Hz, CH_2_*CH*_3_); ^13^C-NMR (300 MHz, DMSO-*d*_6_): 163.1, 160.6, 158.2 and 155.8 (C=O), 148.7, 136.3, 134.7, 130.5 and 124.9 (Ar), 75.2 (C=C), 35.7 (*C*H_2_CH_3_), 21.0 (ArCH_3_), 13.1 (CH_2_*C*H_3_); LC-MS (ESI-ve) Found [M − H]^−^ 312.00 (C_16_H_1__4_N_3_O_4_ requires 312.10).

*3-Ethyl-5-((4-(trifluoromethyl)phenyl)amino)-1H-pyrano[2,3-d]pyrimidine-2,4,7(3H)-trione* (**9f**).

Yield: 63%; pale yellow crystals, no clear melting point (decomposed); ^1^H-NMR (400 MHz, DMSO-*d*_6_): 13.10 (s, 1H, NH), 10.40 (s, 1H, NH), 7.26 (m, 4H, ArH), 4.84 (s, 1H, C=CH), 3.86 (m, 2H, *CH*_2_CH_3_), 1.15 (m, 3H, CH_2_*CH*_3_); ^13^C-NMR (300 MHz, DMSO-*d*_6_): 163.1, 160.6, 158.2 and 155.8 (C=O), 155.9, 125.0 (Ar), 75.2 (C=C), 35.7 (*C*H_2_CH_3_), 13.1 (CH_2_*C*H_3_); LC-MS (ESI-ve) Found [M − H]^−^ 366.00 (C_16_H_1__1_F_3_N_3_O_4_ requires 366.07).

### Appendix C.7. General Procedure G

*3-Amino-3-(2,4,6-trioxotetrahydropyrimidin-5(2H)-ylidene)propanamides***10a**–**q**. A solution of 5-(amino)-2*H*-pyrano[2,3-*d*]pyrimidine-2,4,7(1*H*,3*H*)-trione **9** and amine in 2,2,2-trifluoroethanol was irradiated in a microwave reactor at 150 °C for 1 h. After cooling, the solvent was removed, and acetone was added. The resulting precipitate was filtered off to give pure **10a**–**q**.

*(E/Z)-1-benzyl-5-(1-((4-methoxyphenyl)amino)-3-morpholino-3-oxopropyl-idene)pyrimidine-2,4,6(1H,3H,5H)-trione* (**10a**).

Yield: 20%; white solid, no clear melting point (decomposed); ^1^H-NMR (300 MHz, CDCl_3_): 13.88 and 13.76 (s, 1H, NH, *E,Z*), 8.41 (s, 1H, NH, *E,Z*), 7.37–6.84 (m, 9H, ArH), 5.02 and 4.69 (s, 2H, ArCH_2_, *E,Z*), 3.93 (s, 2H, COCH_2_, *E,Z*), 3.76 (s, 3H, OCH_3_), 3.67–3.35 (m, 8H, morpholine); ^13^C-NMR (300 MHz, CDCl_3_): 169.9, 166.3, 162.0 and 159.5 (C=O), 150.0, 128.4, 128.2, 127.5, 127.2 and 114.7 (Ar), 90.8 (C=C), 65.5 (OCH_2_, morpholine), 55.6 (OCH_3_), 46.5 (NCH_2_, morpholine), 43.6 (CH_2_); LC-MS (ESI-ve) Found [M − H]^−^ 477.20 (C_25_H_25_N_4_O_6_ requires 477.18).

*(E/Z)-1-benzyl-5-(3-morpholino-3-oxo-1-(p-tolylamino)propylidene)pyrimidine-2,4,6(1H,3H,5H)-trione* (**10b**).

Yield: 50%; white solid, no clear melting point (decomposed); ^1^H-NMR (300 MHz, CDCl_3_): 13.96 and 13.82 (s, 1H, NH, *E,Z*), 7.87 (s, 1H, NH, *E,Z*), 7.38–7.10 (m, 9H, ArH), 5.02 and 4.97 (s, 2H, ArCH_2_, *E,Z*), 3.93 (s, 2H, COCH_2_, *E,Z*), 3.67-3.33 (m, 8H, morpholine), 2.32 (s, 3H, ArCH_3_); ^13^C-NMR (500 MHz, CDCl_3_): 170.3, 167.2, 166.3 and 162.5 (C=O), 150.0, 138.7, 137.4, 136.9, 133.2, 130.0, 128.4, 127.5 and 124.7 (Ar), 90.8 (C=C), 66.8 (OCH_2_, morpholine), 46.5 (NCH_2_, morpholine), 42.3 (ArCH_2_), 36.9 (COCH_2_), 21.2 (CH_3_Ar); LC-MS (ESI-ve) Found [M − H]^−^ 461.15 (C_25_H_25_N_4_O_5_ requires 461.18).

*(E/Z)-3-(1-benzyl-2,4,6-trioxotetrahydropyrimidin-5(2H)-ylidene)-3-((4-methoxyphenyl)amino)-N-(4-(trifluoromethyl)phenyl)propenamide* (**10c**).

Yield: 15%; yellow solid, no clear melting point (decomposed); ^1^H-NMR (300 MHz, DMSO-*d*_6_): 14.00 and 13.47 (s, 1H, NH, *E,Z*), 11.36 and 10.98 (s, 1H, NH, *E,Z*), 10.51 and 10.31 (s, 1H, NH, *E,Z*), 7.99–6.53 (m, 13H, ArH), 5.01 and 4.91 (s, 2H, ArCH_2_, *E,Z*), 4.08 and 4.06 (s, 2H, COCH_2_, *E,Z*), 3.77 (s, 3H, OCH_3_); ^13^C-NMR (300 MHz, DMSO-*d*_6_): 164.5 (C=O), 152.6, 127.6, 126.6 and 113.4 (Ar), 92.2 (C=C), 55.9 (OCH_3_), 46.2 (CH_2_); LC-MS (ESI-ve) Found [M − H]^−^ 551.05 (C_28_H_22_F_3_N_4_O_5_ requires 551.15).

*(E/Z)-3-(1-benzyl-2,4,6-trioxotetrahydropyrimidin-5(2H)-ylidene)-N-(4-methoxyphenyl)-3-(phenylamino)propenamide* (**10d**).

Yield: 76%; white solid, no clear melting point (decomposed); ^1^H-NMR (300 MHz, DMSO-*d*_6_): 14.17 and 13.89 (s, 1H, NH, *E,Z*), 11.36 and10.98 (s, 1H, NH, *E,Z*), 9.93 and 9.92 (s, 1H, NH, *E,Z*), 7.49–6.84 (m, 14H, ArH), 5.01 and 4.93 (s, 2H, ArCH_2_, *E,Z*), 4.06 and 4.03 (s, 2H, COCH_2_, *E,Z*); ^13^C-NMR (300 MHz, DMSO-*d*_6_): 167.3, 162.8 and 155.6 (C=O), 150.2, 135.0, 132.5, 130.1, 128.8, 127.5, 126.3, 120.9 and 114.2 (Ar), 92.2 (C=C), 55.6 (OCH_3_), 43.2 (CH_2_); LC-MS (ESI-ve) Found [M − H]^−^ 483.15 (C_27_H_23_N_4_O_5_ requires 483.17).

*(E/Z)-3-(1-benzyl-2,4,6-trioxotetrahydropyrimidin-5(2H)-ylidene)-N-(4-chlorophenyl)-3-(phenylamino)propenamide* (**10e**).

Yield: 50%; white solid, no clear melting point (decomposed); ^1^H-NMR (300 MHz, DMSO-*d*_6_): 14.16 and 13.88 (s, 1H, NH, *E,Z*), 11.38 and 10.99 (s, 1H, NH, *E,Z*), 10.24 and 10.26 (s, 1H, NH, *E,Z*), 7.51–7.22 (m, 14H, ArH), 5.01 and 4.92 (s, 2H, ArCH_2_, *E,Z*), 4.06 and 4.04 (s, 2H, COCH_2_, *E,Z*); ^13^C-NMR (300 MHz, DMSO-*d*_6_): 167.3, 166.4, 166.3 and 162.8 (C=O), 150.3, 138.3, 136.2, 129.0, 128.6, 127.7, 127.5, 126.3 and 120.9 (Ar), 91.18 (C=C), 43.2 (CH_2_); LC-MS (ESI-ve) Found [M − H]^−^ 487.15 (C_26_H_20_^35^ClN_4_O_4_ requires 487.12).

*(E/Z)-3-(1-ethyl-2,4,6-trioxotetrahydropyrimidin-5(2H)-ylidene)-3-((4-methoxyphenyl)amino)-N-(p-tolyl)propenamide* (**10f**).

Yield: 30%; white crystals, no clear melting point (decomposed); ^1^H-NMR (400 MHz, DMSO-*d*_6_): 13.98 and 13.85 (s, 1H, NH, *E,Z*), 11.23 and 10.84 (s, 1H, NH, *E,Z*), 9.99 and 9.96 (s, 1H, NH, *E,Z*), 7.44–6.87 (m, 8H, ArH), 4.04 and 4.02 (s, 2H, COCH_2_, *E,Z*), 3.83 and 3.81 (m, 2H, *CH*_2_CH_3_), 3.76 (s, 3H, OCH_3_), 2.24 (s, 3H, ArCH_3_), 1.15 and 1.05 (t, *J* = 7.0 Hz, 3H, CH_2_*CH*_3_); ^13^C-NMR (400 MHz, DMSO-*d*_6_): 169.7, 166.0, 162.9 and 159.3 (C=O), 150.0, 137.0, 132.4, 130.6, 129.5, 128.8, 119.4 and 115.2 (Ar), 91.2 (C=C), 55.9 (OCH_3_), 35.1 (*CH*_2_CH_3_), 20.9 (ArCH_3_), 13.8 (CH_2_*CH*_3_); LC-MS (ESI-ve) Found [M − H]^−^ 435.15 (C_23_H_23_N_4_O_5_ requires 435.17).

*(E/Z)-N-(4-chlorophenyl)-3-(1-ethyl-2,4,6-trioxotetrahydropyrimidin-5(2H)-ylidene)-3-((4-methoxyphenyl)amino)propenamide* (**10g**).

Yield: 70%; white solid, no clear melting point (decomposed); ^1^H-NMR (400 MHz, DMSO-*d*_6_): 13.98 and 13.84 (s, 1H, NH, *E,Z*), 11.22 and 10.83 (s, 1H, NH, *E,Z*), 10.29 and 10.20 (s, 1H, NH, *E,Z*), 7.55–7.05 (m, 8H, ArH), 4.04 and 4.02 (s, 2H, COCH_2_, *E,Z*), 3.83 and 3.82 (m, 2H, *CH*_2_CH_3_), 3.77 (s, 3H, OCH_3_), 1.13 and 1.04 m, 3H, CH_2_*CH*_3_); ^13^C-NMR (400 MHz, DMSO-*d*_6_): 169.4, 167.2, 162.9 and 159.4 (C=O), 150.0, 138.4, 129.1, 128.8, 127.6, 127.0, 120.9 and 115.3 (Ar), 91.1 (C=C), 55.9 (OCH_3_), 36.8 (*CH*_2_CH_3_), 13.8 (CH_2_*CH*_3_); LC-MS (ESI-ve) Found [M − H]^−^ 455.05 (C_22_H_20_^35^ClN_4_O_5_ requires 455.11).

*(E/Z)-N-(3-chlorophenyl)-3-(1-ethyl-2,4,6-trioxotetrahydropyrimidin-5(2H)-ylidene)-3-((4-methoxyphenyl)amino)propenamide* (**10h**).

Yield: 52%; white crystals, melting point 258–261 °C; ^1^H-NMR (400 MHz, DMSO-*d*_6_): 13.98 and 13.84 (s, 1H, NH, *E,Z*), 11.21 and 10.82 (s, 1H, NH, *E,Z*), 10.33 and 10.30 (s, 1H, NH, *E,Z*), 7.76 – 6.88 (m, 8H, ArH), 4.04 and 4.02 (s, 2H, COCH_2_, *E,Z*), 3.86 and 3.85 (m, 2H, *CH*_2_CH_3_), 3.77 (s, 3H, OCH_3_), 1.14 and 1.04 (m, 3H, CH_2_*CH*_3_); ^13^C-NMR (400 MHz, DMSO-*d*_6_): 169.4, 167.2, 166.7 and 162.9 (C=O), 150.0, 140.9, 133.5, 130.9, 128.8, 123.2, 117.7 and 115.3 (Ar), 91.2 (C=C), 55.9 (OCH_3_), 34.9 (*CH*_2_CH_3_), 13.8 (CH_2_*CH*_3_); LC-MS (ESI-ve) Found [M − H]^−^ 455.05 (C_22_H_20_^35^ClN_4_O_5_ requires 455.11).

*(E/Z)-N-(2-chlorophenyl)-3-(1-ethyl-2,4,6-trioxotetrahydropyrimidin-5(2H)-ylidene)-3-((4-methoxyphenyl)amino)propenamide* (**10i**).

Yield: 55%; white crystals, melting point 266–269 °C; ^1^H-NMR (400 MHz, DMSO-*d*_6_): 13.96 and 13.83 (s, 1H, NH, *E,Z*), 11.20 and 10.85 (s, 1H, NH, *E,Z*), 9.67 and 9.63 (s, 1H, NH, *E,Z*), 7.69–7.06 (m, 8H, ArH), 4.14 and 4.12 (s, 2H, COCH_2_, *E,Z*), 3.86 and 3.85 (m, 2H, *CH*_2_CH_3_), 3.79 (s, 3H, OCH_3_), 1.15 and 1.05 (m, 3H, CH_2_*CH*_3_); ^13^C-NMR (400 MHz, DMSO-*d*_6_): 169.5, 167.2, 163.0 and 159.4 (C=O), 150.0, 135.3, 129.9, 128.8, 126.6 and 115.2 (Ar), 91.2 (C=C), 55.9 (OCH_3_), 35.1 (*CH*_2_CH_3_), 13.8 (CH_2_*CH*_3_); LC-MS (ESI-ve) Found [M − H]^−^ 455.05 (C_22_H_20_^35^ClN_4_O_5_ requires 455.11).

*(E/Z)-3-(1-ethyl-2,4,6-trioxotetrahydropyrimidin-5(2H)-ylidene)-3-((4-methoxyphenyl)amino)-N-(4-(trifluoromethyl)phenyl)propenamide* (**10j**).

Yield: 73%; white crystals, no clear melting point (decomposed); ^1^H-NMR (400 MHz, DMSO-*d*_6_): 13.98 and 13.84 (s, 1H, NH, *E,Z*), 11.22 (s, 1H, NH, *E,Z*), 10.84 and 10.51 (s, 1H, NH, *E,Z*), 7.72–6.62 (m, 8H, ArH), 4.07 (s, 2H, COCH_2_, *E,Z*), 3.86 (m, 2H, *CH*_2_CH_3_, *E,Z*), 3.77 (s, 3H, OCH_3_, *E,Z*), 1.14 and 1.03 (m, 3H, CH_2_*CH*_3_); LC-MS (ESI-ve) Found [M − H]^−^ 489.15 (C_23_H_20_F_3_N_4_O_5_ requires 489.14).

*(E/Z)-3-(1-ethyl-2,4,6-trioxotetrahydropyrimidin-5(2H)-ylidene)-N-phenyl-3-(p-tolylamino)propanamide* (**10k**).

Yield: 73%; white solid, no clear melting point (decomposed); ^1^H-NMR (300 MHz, DMSO-*d*_6_): 14.06 and 13.92 (s, 1H, NH, *E,Z*), 11.22 and 10.84 (s, 1H, NH, *E,Z*), 10.11 and 10.09 (s, 1H, NH, *E,Z*), 7.51–7.05 (m, 8H, ArH), 4.05 and 4.04 (s, 2H, COCH_2_, *E,Z*), 3.86 and 3.75 (m, 2H, *CH*_2_CH_3_), 2.32 (s, 3H, ArCH_3_), 1.14 and 1.036 (m, 3H, CH_2_*CH*_3_); ^13^C-NMR (300 MHz, DMSO-*d*_6_): 168.9, 167.3, 166.2 and 162.8 (C=O), 150.0, 139.5, 138.4, 133.7, 130.6, 129.1, 126.3, 123.5 and 119.3 (Ar), 91.4 (C=C), 35.1 (*CH*_2_CH_3_), 21.1 (ArCH_3_), 13.8 (CH_2_*CH*_3_); LC-MS (ESI-ve) Found [M − H]^−^ 405.10 (C_22_H_21_N_4_O_4_ requires 405.16).

*(E/Z)-3-(1-ethyl-2,4,6-trioxotetrahydropyrimidin-5(2H)-ylidene)-N-(4-methoxyphenyl)-3-(p-tolylamino)propanamide* (**10l**).

Yield: 67%; white solid, no clear melting point (decomposed); ^1^H-NMR (300 MHz, DMSO-*d*_6_): 14.06 and 13.92 (s, 1H, NH, *E,Z*), 11.21 and 10.82 (s, 1H, NH, *E,Z*), 9.95 and 9.91 (s, 1H, NH, *E,Z*), 7.40–6.85 (m, 8H, ArH), 4.03 and 4.02 (s, 2H, COCH_2_, *E,Z*), 3.85 and 3.83 (m, 2H, *CH*_2_CH_3_), 3.71 (ArOCH_3_), 2.32 (s, 3H, ArCH_3_), 1.14 and 1.04 (m, 3H, CH_2_*CH*_3_); ^13^C-NMR (300 MHz, DMSO-*d*_6_): 167.3, 165.7, 162.9 and 155.6 (C=O), 150.0, 138.3, 133.7, 130.6, 126.0, 120.9 and 114.3 (Ar), 91.2 (C=C), 55.6 (ArOCH_3_), 35.0 (*CH*_2_CH_3_), 21.1 (ArCH_3_), 13.8 (CH_2_*CH*_3_); LC-MS (ESI-ve) Found [M − H]^−^ 435.10 (C_23_H_23_N_4_O_5_ requires 435.17).

*(E/Z)-N-(4-chlorophenyl)-3-(1-ethyl-2,4,6-trioxotetrahydropyrimidin-5(2H)-ylidene)-3-(p-tolylamino)propanamide* (**10m**).

Yield: 49%; white crystals, no clear melting point (decomposed); ^1^H-NMR (300 MHz, DMSO-*d*_6_): 14.06 and 13.92 (s, 1H, NH, *E,Z*), 11.22 and 10.83 (s, 1H, NH, *E,Z*), 10.29 and 10.25 (s, 1H, NH, *E,Z*), 7.55–7.23 (m, 8H, ArH), 4.04 and 4.02 (s, 2H, COCH_2_, *E,Z*), 3.84 and 3.73 (q, 2H, *CH*_2_CH_3_), 2.32 (s, 3H, ArCH_3_), 1.14 and 1.03 (t, 3H, CH_2_*CH*_3_); ^13^C-NMR (400 MHz, DMSO-*d*_6_): 169.2, 167.3, 162.9 and 155.0 (C=O), 150.0, 138.4, 133.7, 130.6, 129.1, 126.0, 120.9 and 119.4 (Ar), 91.2 (C=C), 35.1 = (*CH*_2_CH_3_), 211 (ArCH_3_), 13.8 (CH_2_*CH*_3_); LC-MS (ESI-ve) Found [M − H]^−^ 439.05 (C_22_H_20_^35^ClN_4_O_4_ requires 439.12).

*(E/Z)-N-(3-chlorophenyl)-3-(1-ethyl-2,4,6-trioxotetrahydropyrimidin-5(2H)-ylidene)-3-(p-tolylamino)propenamide* (**10n**).

Yield: 63%; white crystals, melting point 284–285 °C; ^1^H-NMR (400 MHz, DMSO-*d*_6_): 14.063 and 13.92 (s, 1H, NH, *E,Z*), 11.23 and 10.84 (s, 1H, NH, *E,Z*), 10.34 and 10.30 (s, 1H, NH, *E,Z*), 7.75–7.10 (m, 8H, ArH), 4.04 and 4.02 (s, 2H, COCH_2_, *E,Z*), 3.86 and 3.75 (m, 2H, *CH*_2_CH_3_), 2.32 (s, 3H, ACH_3_), 1.16 and 1.02 (m, 3H, CH_2_*CH*_3_); ^13^C-NMR (400 MHz, DMSO-*d*_6_): 169.0, 168.5, 166.6 and 162.9 (C=O), 150.0, 140.9, 138.4, 133.5, 130.9, 126.1, 123.2, 118.8 and 117.7 (Ar), 91.2 (C=C), 35.1 (*CH*_2_CH_3_), 21.1 (ArCH_3_), 13.8 (CH_2_*CH*_3_); LC-MS (ESI-ve) Found [M − H]^−^ 439.05 (C_22_H_20_^35^ClN_4_O_4_ requires 439.12).

*(E/Z)-3-(1-ethyl-2,4,6-trioxotetrahydropyrimidin-5(2H)-ylidene)-N-(4-methoxyphenyl)-3-((4-(trifluoromethyl)phenyl)amino)propanamide* (**10o**).

Yield: 44%; grey solid, no clear melting point (decomposed); ^1^H-NMR (400 MHz, DMSO-*d*_6_): 14.27 and 14.10 (s, 1H, NH, *E,Z*), 11.51 (s, 1H, NH, *E,Z*), 10.01 and 9.97 (s, 1H, NH, *E,Z*), 7.90–6.51 (m, 8H, ArH), 4.08 (s, 2H, COCH_2_, *E,Z*), 3.86 and 3.85 (m, 2H, *CH*_2_CH_3_, *E,Z*), 3.79-3.77 (s, 3H, OCH_3_, *E,Z*), 1.15 and 1.05 (m, 3H, CH_2_*CH*_3_); ^13^C-NMR (400 MHz, DMSO-*d*_6_): 168.7, 167.3, 166.0 and 162.8 (C=O), 150.0, 140.2, 136.9, 132.5, 130.6, 129.5, 127.2, 126.1, 119.5 and 114.5 (Ar), 91.2 (C=C), 55.9 (OCH_3_), 35.1 (*CH*_2_CH_3_), 13.8 (CH_2_*CH*_3_); LC-MS (ESI-ve) Found [M − H]^−^ 489.05 (C_23_H_20_F_3_N_4_O_5_ requires 489.14).

*(E/Z)-3-(1-ethyl-2,4,6-trioxotetrahydropyrimidin-5(2H)-ylidene)-N-(p-tolyl)-3-((4-(trifluoromethyl)phenyl)amino)propenamide* (**10p**).

Yield: 40%; white solid, no clear melting point (decomposed); ^1^H-NMR (300 MHz, DMSO-*d*_6_): 14.27 and 14.10 (s, 1H, NH, *E,Z*), 11.32 and 10.92 (s, 1H, NH, *E,Z*), 10.07 and 10.03 (s, 1H, NH, *E,Z*), 7.90-6.46 (m, 8H, ArH), 4.09 (s, 2H, COCH_2_, *E,Z*), 3.87 and 3.76 (m, 2H, *CH*_2_CH_3_), 2.25 (s, 3H, ArCH_3_), 1.15 and 1.05 (m, 3H, CH_2_*CH*_3_, *E,Z*); ^13^C-NMR (400 MHz, DMSO-*d*_6_): 168.7, 167.3, 166.0 and 162.8 (C=O), 150.0, 140.2, 136.9, 132.5, 130.6, 129.5, 127.2, 126.1, 119.5 and 114.5 (Ar), 35.1 (*CH*_2_CH_3_), 20.9 (CH_3_Ar), 13.8 (CH_2_*CH*_3_); LC-MS (ESI-ve) Found [M − H]^−^ 473.1 (C_23_H_20_F_3_N_4_O_4_ requires 473.14).

*(E/Z)-N-(4-chlorophenyl)-3-(1-ethyl-2,4,6-trioxotetrahydropyrimidin-5(2H)-ylidene)-3-((4-(trifluoromethyl)phenyl)amino)propenamide* (**10q**).

Yield: 32%; white solid, no clear melting point (decomposed); ^1^H-NMR (300 MHz, DMSO-*d*_6_): 14.14 and 14.09 (s, 1H, NH, *E,Z*), 10.93 and 10.80 (s, 1H, NH, *E,Z*), 10.30 and 10.28 (s, 1H, NH, *E,Z*), 7.88-6.54 (m, 8H, ArH), 4.08 (s, 2H, COCH_2_, *E,Z*), 3.85 and 3.76 (m, 2H, *CH*_2_CH_3_), 1.14 and 1.05 (m, 3H, CH_2_*CH*_3_); LC-MS (ESI-ve) Found [M − H]^−^ 493.05 (C_22_H_1__7_^35^ClF_3_N_4_O_4_ requires 493.09).
